# Examining the Link Between Implementation Fidelity, Quality, and Effectiveness of Teacher-Delivered Anti-Bullying Interventions in a Randomized Controlled Trial

**DOI:** 10.1007/s11121-023-01580-8

**Published:** 2023-09-01

**Authors:** Chloé Tolmatcheff, René Veenstra, Isabelle Roskam, Benoit Galand

**Affiliations:** 1 Department of Sociology, University of Groningen, Grote Rozenstraat 31, Groningen, 9712 TG the Netherlands; 2 Psychological Sciences Research Institute, University of Louvain, Ottignies-Louvain-la-Neuve, Belgium

**Keywords:** School bullying, Implementation, Fidelity, Quality, Teacher

## Abstract

This study examined the relation between both implementation fidelity and quality and the outcomes of two different anti-bullying interventions targeting distinct processes involved in bullying: moral disengagement and social norms. In total, 34 French-speaking Belgian teachers from six elementary schools were trained to deliver either the moral disengagement or the social norms intervention to their Grade 4–6 students (*N* = 747, 50.4% boys) in a randomized controlled trial. Students reported their moral disengagement, perceived injunctive class norm toward bullying, and bullying behaviors in the fall and spring of the 2018–2019 school year. Teachers’ implementation fidelity and quality were assessed through direct observation in each class by two independent raters with a satisfying interrater reliability. A multiplicative moderated mediation model using latent change scores revealed that both greater fidelity and quality amplified students’ decrease in moral disengagement, which was accompanied by a decrease in bullying. Importantly, when quality was sufficient, fidelity did not matter. However, higher fidelity could compensate for a lack of quality. Contrary to expectations, there was no significant change in students’ perceptions of the class injunctive norm, even under ideal implementation conditions. Alternative mediators should thus be considered. Because implementation by teachers inevitably fluctuates, anti-bullying program components should be evaluated under various implementation conditions to determine their cost-effectiveness ratio and to gain insights into how anti-bullying programs work.

**Supplementary Information:**

The online version contains supplementary material available at 10.1007/s11121-023-01580-8.

Given the high prevalence of bullying in schools and its detrimental consequences for the children involved, an increasing number of anti-bullying programs have been developed over the past three decades (Low et al., 2014). Although anti-bullying programs can be effective to some extent, they do not consistently translate into positive outcomes (Cross & Barnes, 2014). Deviations from intended program implementation may explain the limited and mixed results of anti-bullying programs, especially when programs are delivered by the teachers, which is often the case in bullying prevention (Goncy et al., 2015). Even though implementation strongly influences school-based prevention programs’ outcomes (Durlak & Dupre, 2008), few studies have examined how implementation is associated with anti-bullying program outcomes (Haataja et al., 2014). This was the goal of our study, which examined the influence of implementation in two separate interventions targeting two different processes involved in bullying, namely moral disengagement and social norms, delivered by elementary teachers in a randomized controlled trial.

## Implementation and Anti-Bullying Programs Outcomes

Implementation science is a research area that encompasses various aspects. This paper focuses on the process of delivering the program content from the teachers to the students, which is crucial to interpreting the effects of anti-bullying interventions accurately (Low et al., 2014). A first critical question is whether the specified intervention was correctly carried out. High levels of implementation *fidelity* (the extent to which the delivered intervention corresponds to the prescribed content and procedure; also referred to as adherence or completion) have been linked to positive program outcomes in most studies (Durlak & Dupre, 2008). When it comes to intricate interventions like social-psychological-behavioral interventions, the effectiveness of the program may not solely depend on fidelity but also on the *quality* (how well the program is delivered) of implementation (e.g., Berkel et al., 2011; Schulte et al., 2009). In practice, however, implementation quality is rarely assessed in anti-bullying intervention studies (Tolmatcheff, 2021). Assessing both features fits recommendations from the implementation literature (e.g., Berkel et al., 2011; Goncy et al., 2015).

The findings of the few evaluation studies of anti-bullying programs examining how implementation fidelity and quality relate to outcomes are mixed overall (see Tolmatcheff, 2021 for a review). A possible explanation for this variability is that implementation features may interact in influencing outcomes (Berkel et al., 2011). For instance, the beneficial impact of high fidelity may be contingent upon teachers delivering the intervention in a clear, understandable, and enthusiastic manner (Berkel et al., 2011). While limited, findings from other fields provide support for the idea of an interaction between fidelity and quality. For instance, research in drug counseling has shown that the therapeutic alliance moderates the relation between fidelity and outcomes (Barber et al., 2006). Likewise, an intervention study focusing on students’ literacy found an interaction between fidelity and teachers’ instructional quality (Capin et al., 2022).

Another explanation may be that different implementation features are relevant to different interventions (Durlak & Dupre, 2008; Schulte et al., 2009) or that different interventions (or intervention components) may have distinct thresholds that need to be met to achieve their intended effects (Nelson et al., 2012). The specific implementation features that matter and their extent of importance are likely contingent upon the type and nature of the intervention, including its target and format (Schulte et al., 2009).

How implementation features have been defined, operationalized, and assessed varies among studies (Berkel et al., 2011; Durlak & Dupre, 2008; Schulte et al., 2009). In evaluations of anti-bullying programs, *fidelity* is usually self-reported by the teachers through checklists of activities or elements of the program or specific lessons (Tolmatcheff, 2021). Observational data are, however, more reliable than self-reports and recommended if possible (Durlak & Dupre, 2008). *Quality* includes not only technical but also relational dimensions (Goncy et al., 2015; Haataja et al., 2014) such as classroom management and emotional tone, and sometimes student responsiveness, as reflecting teachers’ ability to engage students (e.g., Hirschtein et al., 2007). Adjustments made by teachers (adaptation) to best fit the local context and their students’ needs without threatening the core elements of the intervention can also be seen as contributing to the quality (Cross & Barnes, 2014; Goncy et al., 2015; Quinn & Kim, 2017; Schulte et al., 2009).

Overall, examining the combined association of different implementation features with the outcomes of different anti-bullying components, using high-quality data collection methods, research designs, and analytical strategies is a crucial avenue for advancing the research agenda in bullying prevention (Berkel et al., 2011).

## Moral Disengagement and Social Norms as Targets of Anti-Bullying Program Components

Our study is part of a larger research project aiming to “open the black box of anti-bullying programs.” The central premise is that the limited effectiveness of anti-bullying programs may be due to the choice of program components—the “ingredients”—as well as three gaps in the evaluation studies: (1) the different program components are usually assessed as a whole package rather than tested separately (Menesini & Salmivalli, 2017); (2) the hypothesized mediating paths are not tested (Saarento et al., 2015); (3) implementation is not taken into account (Haataja et al., 2014). Whereas we addressed the first two gaps in a previous study (Tolmatcheff et al., 2022a), this paper focuses on the third gap. With respect to the “ingredients,” we aimed to evaluate the effects of two different components targeting distinct processes involved in bullying, selected on the basis of prior research and theory: *moral disengagement* and *social norms* (e.g., Hymel & Bonanno, 2014; Menesini et al., 2015).

Bandura’s model of moral disengagement (1986) is based on the idea that individuals can selectively disengage from the moral principles that normally guide their behavior. Transgressing these principles leads to self-sanctions such as guilt and shame. However, using cognitive processes to reinterpret the situation (the so-called moral disengagement mechanisms), one can behave in an immoral way without undergoing self-sanctions. Moral disengagement significantly predicts bullying in both cross-sectional (see Killer et al., 2019 for a meta-analysis) and longitudinal studies (see Thornberg, 2023 for a recent meta-analysis), and is a promising avenue for prevention (e.g., Hymel & Bonanno, 2014; Wang & Goldberg, 2017). If students can no longer use moral disengagement mechanisms, they should no longer be able to deactivate internal controls when transgressing their moral principles. As a result, they will undergo the unpleasant self-sanctions that they were trying to escape, which should in turn discourage their immoral behavior.

Social norms, referring to what is typical or appropriate in a group or situation, are the second explanatory framework for the paradox between students’ private attitudes toward bullying and their actual behavior (Prentice & Miller, 1996). People tend to conform to what they perceive to be the norm over and above their personal attitudes (Veenstra & Lodder, 2022). However, their perceptions are not necessarily accurate. Students tend to overestimate, sometimes drastically, the prevalence of risky or problem behaviors and attitudes among their peers, notably regarding bullying (Dillon & Lochman, 2019; Perkins et al., 2011; Sandstrom et al., 2013). Social norms influence students’ behavior in bullying, regardless of their private attitudes (e.g., Salmivalli & Voeten, 2004; Sandstrom et al., 2013). As a result, most students privately disapprove of bullying but believe that their peers approve of it, that is, a situation of *pluralistic ignorance* (Miller & Prentice, 2016). This biased norm, in turn, enhances bullying as students mistakenly feel support or even social pressure to engage in it (Perkins et al., 2011; Sandstrom et al., 2013). Providing students with correct information about their classmates’ actual attitudes toward bullying can rectify their perceptions of the norm, making bullying less desirable, which is likely to induce a change of behavior (Miller & Prentice, 2016). Thus, correcting students’ misperceptions of the injunctive class norm toward bullying should discourage bullying behaviors.

## Teacher Training and Interventions

More information on the training and intervention content, outlines, and rationales can be found as supplemental materials. The French version of all intervention materials provided to the teachers, including a detailed description of each session and how it unfolds, can be found on OSF (https://osf.io/w5hd7/?view_only=693d6d86743d4c49980a7768ce1e555b). Each class teacher attended a training day delivered between January and February 2019 and was provided with detailed lesson plans and required materials for the activities. Five lessons were delivered by the class teachers to their students for 1 hr a week after the training. We used an active control group, in which teachers implemented an intervention of similar duration and intensity but on a topic unrelated to bullying (climate and environmental issues). This allowed us to control for changes stemming from non-specific treatment factors such as an improvement of relationships between teachers due to the project collaboration, which can lead to spurious intervention effects (Lilienfeld et al., 2014).

The moral disengagement intervention was based on the Bullying Literature Project – Moral Disengagement version (BLP-MD), which uses stories and writing activities to prevent bullying among elementary children (Wang & Goldberg, 2017). We created stories adapted to our sample’s age (available in English as supplemental materials), which represented a variety of bullying situations and illustrated the use of moral disengagement mechanisms by the bullies. The mechanisms were invalidated by the teachers by pointing out to the students that they are false excuses that do not make bullying more acceptable.

The social norms intervention was inspired by the Survey of Bullying at Your School project, which uses students’ self-reported data to reveal the discrepancy between the perceived and actual norms about bullying, and involves social norm messages displayed on posters (Perkins et al., 2011). We helped students to discover their norm misperceptions in an entertaining and instant format suited to their age. Students had to create the social norm messages by themselves, to involve them more closely in the intervention.

## The Present Study

The goal of our study was to examine the relation between implementation fidelity and quality and the outcomes of two different anti-bullying components delivered by elementary teachers within separate interventions in a randomized controlled trial. The two interventions exclusively targeted either moral disengagement or the perceived injunctive class norm as a mediator of change in bullying. Although examining the influence of implementation was an aim of our research project, we have first examined the main effects of the two interventions without taking implementation into account (Tolmatcheff et al., 2022a). In that study, we used a parallel mediation model and contrasted each intervention group with the active control group. Intervening on moral disengagement decreased students’ use of moral disengagement mechanisms, which, in turn, decreased bullying behaviors. The social norms intervention did not yield any change in students’ perceptions of the injunctive class norm toward bullying, but had a direct decreasing effect on bullying nevertheless. Implementation was not taken into account in these previous analyses, and fluctuations in implementation by the teachers may have reduced or concealed the potential effect of both interventions under appropriate implementation conditions.

How implementation fidelity and quality are related to change in the targeted mediator for both interventions was the focus of the present study. Therefore, only data from the two intervention conditions were analyzed. This was possible because each intervention targeted a specific mediator and had no unintended effect on the other intervention’s mediator (Tolmatcheff et al., 2022a). So we were able to validly contrast the two conditions with each other in this study to examine the influence of implementation. Dosage was not considered because there was minimal variability (only three teachers out of 34 had delivered less than five sessions of intervention) (Haataja et al., 2014).

Our primary hypothesis was that higher levels of fidelity or quality would be associated with greater change in the outcomes, that is, a greater reduction in moral disengagement (and, indirectly, bullying) in the moral disengagement intervention group, and a greater increase in the perceived anti-bullying injunctive class norm (and, indirectly, a greater reduction in bullying) in the social norms intervention group. Because intervening on social norms did not yield any change in the intended mediator (Tolmatcheff et al., 2022a), we were interested to examine the effects of this intervention under optimal implementation conditions (i.e., high fidelity and high quality). As a secondary hypothesis, we assumed that fidelity and quality would interact (i.e., multiplicative moderation; Hayes, 2018) in influencing change in the outcomes. Finally, we wanted to examine whether the relation between these two implementation features and students’ outcomes would differ between our two interventions. For example, interventions targeting socio-emotional skills might require greater quality from the teacher to involve the students emotionally, whereas interventions with complex information, such as norm-based messages, might require greater fidelity to change the norm correctly.

## Method

### Sampling and Design

**Fig. 1:**
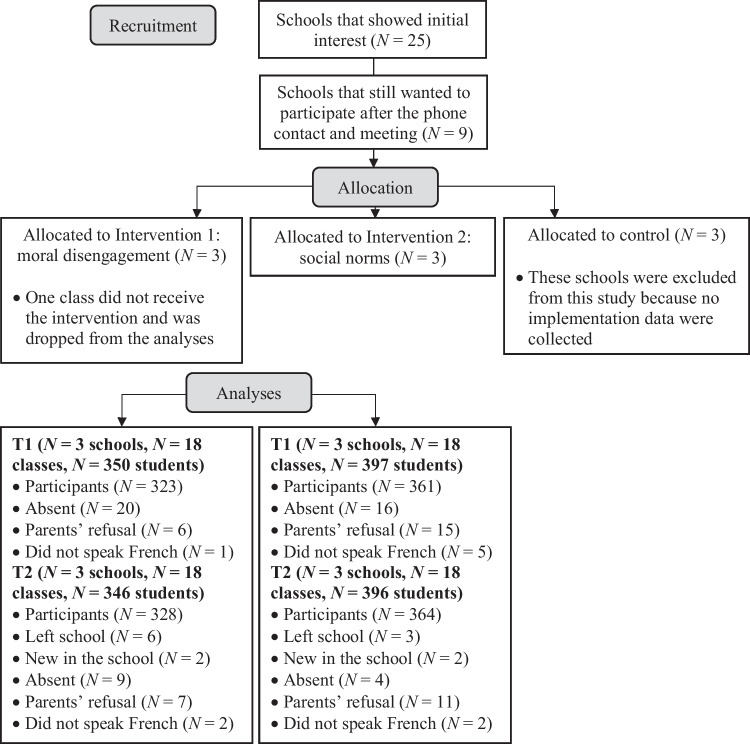
Flowchart of Recruitment and Retention of Intervention and Control Schools. *Note*. Adapted from [“The effectiveness of moral disengagement and social norms as anti-bullying components: A randomized controlled trial”, by Tolmatcheff et al., 2022a, b, *Child Development, 93*, https://doi.org/10.1111/cdev.13828 Copyright ^©^ 2022, by Child Development, Society for Research in Child Development]. Adapted with permission. Modifications made to the original figure: We removed the sample details (lowest boxes, under “Analyses”) for the control group, and added the relevant mention in the “allocated to control” box

A call for recruitment was sent to elementary schools in French-speaking Belgium in the context of a research project on bullying prevention. Schools were recruited on a voluntary basis. The supplemental materials contain detailed information about the power calculation, recruitment, eligibility criteria, randomization process, timeline, data collection, and demographic characteristics of the sample. Nine elementary schools of various size, geographical location, and socioeconomic index agreed to participate in the project and were randomly assigned to either one of the two intervention conditions or the control group. The original sample comprised 1,216 Grade 4–6 students from 57 classes at baseline. Implementation data were collected in intervention schools only (*N* = 6), thus providing a final sample of 747 students for this study (50.4% boys, *M* age = 10.2, *SD* = 1.0). Figure 1 presents a flowchart of the recruitment and retention of intervention and control schools.

### Data Collection and Participants

The data collection took place in the fall of 2018 and the spring of 2019, with an average total of 15 weeks between the two waves. The schedule could vary between schools because of school vacations, school trips, and other appointments, but the time lapse between the end of the intervention and the second wave of data collection was the same for all schools (1–2 weeks). The online supporting information provides a detailed timeline of the data collection. Passive consent forms had been sent to parents prior to data collection. Students did not participate in the event of parental objection or if they themselves did not want to participate. Confidential paper questionnaires were filled in during class hours under the supervision of one or two research team members, who were available to answer questions. The survey was introduced to the students as an “investigation of children’s well-being at school” and a few basic rules were outlined to ensure confidentiality and free participation. The whole procedure was approved by the ethics committee of the research institute of the last author and was in compliance with APA ethical standards. The online supplemental materials contain the CONSORT (Consolidated Standards of Reporting Trials) checklist. At pre-test, the Intervention 1 sample comprised 350 students (49.7% boys, *M* age = 10.13, *SD* = 1.06) and the Intervention 2 sample comprised 397 students (49.1% boys, *M* age = 10.28, *SD* = 0.96).

Implementation data were collected by two raters (the principal researcher and a trained master’s student) who both attended the same key lesson (i.e., lesson 3 for the first intervention and lesson 2 for the second intervention) in each intervention classroom (*N* = 34). All sessions were rated by the principal researcher (first author), who developed the interventions and trained the teachers, and 85% were also independently rated by the master’s student to check the interrater agreement. Once we had ensured that acceptable interrater reliability was achieved for both fidelity and quality (see Measures section), only ratings from the principal researcher were used in the analyses because of her better knowledge and understanding of both the interventions and the theoretical frameworks.

### Measures

#### Implementation Measures

##### Fidelity

We developed a checklist of the core elements in the lesson plan of the specific session that we would observe for both interventions (Hirschstein et al., 2007). Because the two sessions differ, we created 13 items for the moral disengagement and 11 items for the social norms intervention. Items were rated on a 3-point Likert scale, from 0 = *not implemented* to 2 = *fully implemented*. Examples of items are: “The teacher makes the students look for the moral disengagement mechanism(s) used by the bullies in the story” and “The teacher asks the leader of each work group to read aloud the newly created norm-based message to the whole class*.*” Based on the 28 overlapping observations, we computed weighted kappa coefficients to assess the degree of agreement between the two raters (Landis & Koch, 1977). Interrater reliability ranged from substantial (*K* = .75) to almost perfect (*K* = .96) in the moral disengagement intervention, and from moderate (*K* = .56) to almost perfect (*K* = 1) in the social norms intervention (Landis & Koch, 1977). Following recommendations for estimating reliability coefficients with ordinal Likert-type scales, we used the polychoric correlation matrix to compute an ordinal Cronbach’s alpha (Gadermann et al., 2012) for each intervention: *α* = .75 for the moral disengagement and *α* = .83 for the social norms intervention.

##### Quality of Delivery

We used eight items to assess four aspects of quality (Hirschstein et al., 2007): formal aspects of the delivery (clarity, fluency), classroom management (discipline, supervision), relational aspects (listening, not judging), and ability to stimulate students’ engagement (interest, involvement). For instance, “The teacher respects students’ opinions and does not make value judgements.” A ninth item related to teachers’ ability to adapt the lesson to their students’ needs, that is, small adjustments that do not jeopardize the lesson’s core elements. Items were rated on a 3-point Likert scale, from 0 = *poor* to 2 = *excellent*. Interrater reliability (based on the 28 overlapping observations) ranged from moderate (*K* = .53) to substantial (*K* = .71) (Landis & Koch, 1977). As this measure of quality is supposed to be independent of the specific intervention content, we computed a common ordinal alpha for both interventions, which was *α* = .87.

#### Student Outcomes

##### Moral Disengagement

We used the French version of the Moral Disengagement in Bullying Scale (Tolmatcheff et al., 2022b), adapted from Thornberg and Jungert (2014). In the present study, we used only 14 of the 18 items (that is, two items per mechanism) to shorten the questionnaire. Students were asked to indicate to what degree they agreed or disagreed with each statement (from 0 = *totally disagree* to 4 = *totally agree*), e.g., “If people are weird, it is their own fault if they get bullied.” Ordinal alpha was *α* = .84 at T1 and .87 at T2. The result of the confirmatory factorial analysis (CFA) showed a good fit for the higher-order structure: χ^2^(70, *N* = 1094) = 120.7, *p* < .001, CFI = .97, RMSEA = .026, 95% CI = .018–.033, and SRMR = .027. Because of a small negative residual variance for one of the first-order factors (advantageous comparison) at T2, this factor variance was fixed as a small positive value (0.1) to avoid convergence problems. Based on the modification indices, we also allowed one pair of items to correlate because of parallel wording within the same factor (Morin et al., 2016).

##### Perceived Injunctive Anti-Bullying Class Norm

We translated into French eight items from Salmivalli and Voeten (2004) measuring attitudes toward bullying, e.g., “Bullying may be fun sometimes” (from 0 = *totally disagree* to 4 = *totally agree*). We followed the original Survey of Bullying at Your School project procedure for measuring the perceived norms toward bullying and asked the students to indicate what they thought that most of their classmates would answer, instead of their own opinion (Perkins et al., 2011). Two items were deleted because they were too difficult for the students to understand. The six remaining items had ordinal Cronbach’s alphas of *α* = .78 at T1 and *α* = .80 at T2. As four of the six items were reversed, a method-specific factor was added to the measurement model of the scale to take account of this shared specific variance (Geiser, 2013; Morin et al., 2016). The CFA result showed a good fit: χ^2^(5, *N* = 1091) = 9.5, *p* = .089, CFI = .99, RMSEA = .029, 95% CI = .000–.056, and SRMR = .013.

##### Bullying

A 10-item scale intended for primary school students (Tolmatcheff et al., 2020) assessed direct (3 items: insulting; hitting or kicking or slapping; biting or scratching or pulling hair), indirect (4 items: excluding; gossiping; hiding or damaging personal things; teasing), and cyber (3 items: insulting or intimidating through the Internet; calling or texting; releasing pictures or videos) bullying behaviors. Students had to indicate how often (from 0 to 4 = *4 times or more*) they had engaged in each behavior toward another student over the last three months e.g., “I have hidden or damaged another student’s things on purpose.” We used a bifactor model to assess both the general construct of bullying shared by the facets and the specific facets (indirect, direct, and cyber) simultaneously (Morin et al., 2016). The CFA demonstrated a good fit: χ^2^(45, *N* = 1097) = 34.8, *p* = .21, CFI = .99, RMSEA = .014, 95% CI = .000–.028, and SRMR = .025. Ordinal Cronbach’s alphas were *α* = .85 (T1) and *α* = .83 (T2) for the global construct, *α* = .74 (T1) and *α* = .75 (T2) for indirect bullying, *α* = .81 (T1) and *α* = .81 (T2) for direct bullying, and *α* = .85 (T1) and *α* = .84 (T2) for cyber bullying.

### Analytical Strategy

The analyses were conducted in MPlus 8.4. The intraclass coefficients (ICCs) for bullying, moral disengagement, and perceived injunctive class norm were respectively .058, .030, and .037 at the class level at pre-test. Because our interest lays in implementation discrepancies between teachers and the ICCs at the school level were low (a separate analysis showed that .029, .005, and .006 of the variance was at the school level), only the class level was taken into account in the analysis. Power analysis, conducted in Optimal Design (Raudenbush et al., 2011), indicated that a two-level cluster RCT design with 34 clusters and the aforementioned portions of explained variance at the classroom level leads to a power of .80 to detect an effect size of .25 for a significance level of *α* = .10 (see supplementary materials). MLR (maximum likelihood robust) is the default estimation with complex survey data in this case and provides parameter estimates which are robust to non-normality and non-independence of observations. MLR is also a leading technique for handling missing data, using full-information maximum likelihood (FIML) (Geiser, 2013). In the final model, data were missing on all observed dependent variables in 18 cases (2.6%), which Mplus automatically excluded from the analysis. The covariance coverage of data was above 92% for all the indicators.

We used latent change (LC) models to model change between the pre- and post-test of both mediators and bullying. LC models rely on the decomposition of the latent state variable at Time 2 (State 2) into initial state (State 1) plus change, that is, the latent difference variable (State 2 – State 1; see Fig. 3) (Geiser, 2013). Because indicators share a specific variance with themselves over time in longitudinal analyses, the assumption of uncorrelated residuals is likely to be violated. To avoid this issue, we allowed the residuals corresponding to the same item to correlate over time (Geiser, 2013).

We first tested measurement invariance across time for all endogenous variables, as LC models require at least strong factorial invariance. We progressively constrained factor loadings, intercepts, and residual variances to be equal, and tested the fit of each nested model against the less restrictive one (Geiser, 2013). Weak non-invariance is indicated by a CFI difference greater than .01 in combination with a difference in the RMSEA greater than .015 or a difference in SRMR greater than .03. The same applies to strong non-invariance, with the exception that the difference in SRMR should be greater than .01 (Morin et al., 2016).

**Fig. 2:**
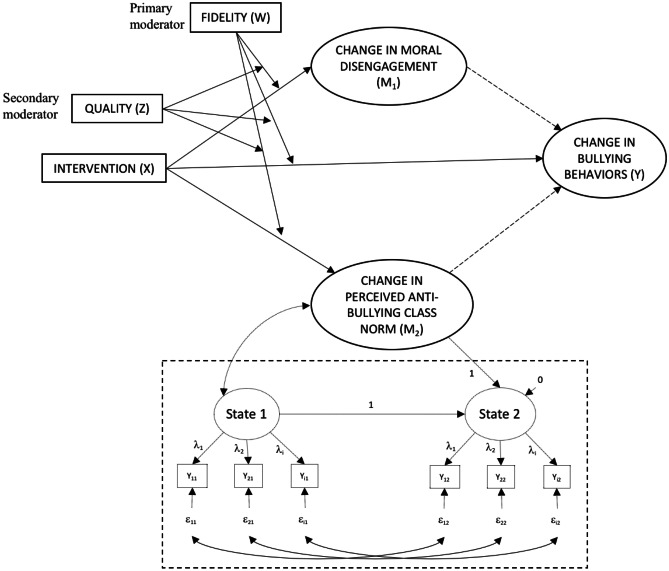
Conceptual diagram of the moderated mediation model. Note: Each latent variable (indicated by an ellipse) displayed on the statistical diagram implies a latent change model with autocorrelated errors. For the sake of clarity, the LC model of perceived injunctive anti-bullying class norm is presented, whereas similar LC models of moral disengagement and bullying are hidden

**Fig. 3:**
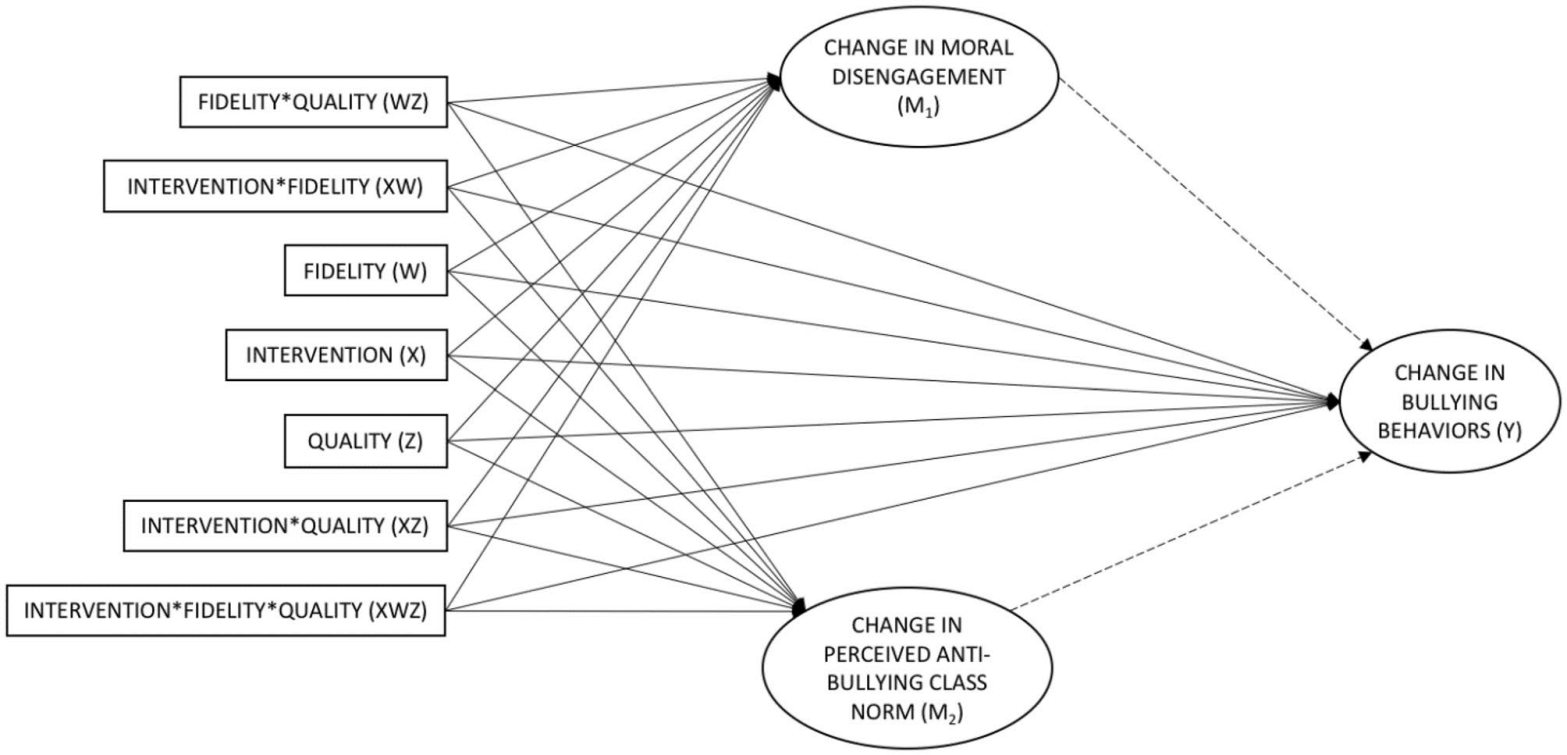
Statistical diagram of the moderated mediation model

Next, we used a moderated mediation model to examine how fidelity and quality related to the outcomes of interest. Figure 2 represents this model in the form of a conceptual diagram. Both moderators were standardized prior to the analysis—fidelity was standardized in each intervention group separately. The statistical diagram (see Fig. 3) illustrates the three-way interaction between fidelity, quality, and the intervention, and the six conditional effects and interactions of these three variables, which should be included even if they are not statistically significant (Hayes, 2018). Importantly, their regression coefficients are conditioned on the other variables being zero (Hayes, 2018). We tested the significance of the three-way interaction term for the effect of X (intervention) on both mediators, as well as on the direct effect on bullying. As our teacher sample size was relatively small (*N* = 34) and we might lack statistical power to test the relevant interaction effects, we set the significance level to *α* = .10. A significant three-way interaction term indicates that the effect of X varies across levels of Z, W, and/or the interaction of Z and W (Hayes, 2018). In case of a non-significant three-way interaction term, we removed it and tested whether the two-way interaction terms were significant. If not, we removed them as well and tested for the significance of the main effects of fidelity and quality.

Next, we used a pick-a-point approach to probe the significant interactions as evidence of moderation does not inform us about where in the distribution the moderator has an effect that is different from zero and where it does not (Hayes, 2018). We selected the values of the mean (0) and a standard deviation below (− 1) and above the mean (+ 1) for both fidelity and quality (Hayes, 2018). Then, we estimated the conditional effects for the nine possible combinations of fidelity and quality levels, and required an inferential test for each combination. In the case of a significant interaction effect for a mediator, we also estimated the indirect effect of X on bullying flowing through the relevant mediator for all the possible combinations. In an additional analysis, we estimated the indirect effect of X on indirect, direct, and cyberbullying where appropriate (see supplementary materials).

All effects were contrasted with the other intervention condition and thus reflect average differences between the two groups. For instance, a significant three-way interaction in a mediator means that the average difference between the two intervention groups in this mediator depends on W and/or Z. To help visualize and interpret the influence of the moderators in a group independently, we plotted the absolute expected values of the relevant mediator for the relevant group at various levels of fidelity and quality (Hayes, 2018). Finally, to go beyond face validity, we requested inferential tests of the slope differences that could be observed on the plots. To do so, we used either high (+ 1 SD) or low (− 1 SD) levels of fidelity or quality as the reference for the statistical significance of conditional effects provided by Mplus.

### Transparency and Openness

We report how we determined our sample size, all data exclusions, and all manipulations in the study (see also supplemental materials). This study is part of a larger research project including several different studies. As such, the student questionnaire included many measures (e.g., empathy, attitudes toward bullying, assistant, defender, and outsider behavior, peer-reported popularity, acceptance, and rejection) intended for other research questions. None of these other measures has been examined except for bullying, defender and outsider behaviors, which were used as distal outcome variables in Tolmatcheff et al. (2022a). Only bullying was used as a distal outcome variable in the present study given (a) the increased complexity of the statistical model (as multiplicative moderators were added to the mediation model) and the simultaneous reduction of the sample size (as the control group was excluded from the analysis); (b) our primary interest in how implementation was associated with change in the proximal outcomes (i.e., the mediators). This study’s design and its analysis were not preregistered. The data and analysis code for this study can be obtained by emailing the corresponding author. All materials are available at https://osf.io/w5hd7/?view_only=693d6d86743d4c49980a7768ce1e555b.

## Results

Table 1 presents the descriptive statistics and inter-correlations between fidelity and quality for both intervention conditions. On average, teachers in the social norms condition demonstrated higher fidelity in implementing the intervention, achieving scores up to a maximum of 2, indicating “perfect” implementation fidelity. Supplemental materials provide scatterplots of implementation fidelity and quality for each condition. Preliminary analyses revealed moderate to large correlations between fidelity and quality.

**Table 1:** Descriptive statistics and inter-correlations between implementation features

					Percentiles			Correlations	
	*M*	*SD*	Min.	Max.	25	50	75	*r*	*p*-value
Moral disengagement									
Fidelity (*n* = 16)	1.14	0.41	0.46	1.85	0.73	1.23	1.44	.44	.085
Quality (*n* = 16)	1.37	0.49	0.56	2.00	0.86	1.56	1.77	—	
Social norms									
Fidelity (*n* = 17)	1.42	0.44	0.40	2.00	1.25	1.50	1.70	.63	< .01
Quality (*n* = 18)	1.31	0.48	0.44	2.00	0.86	1.33	1.78	—	

Total *N* = 34

We could not assess implementation in two classes from the moral disengagement condition because the teachers were absent on the day of observation. Because of an agenda problem, one teacher in the social norms condition delivered a session other than the planned one. We were therefore unable to observe fidelity for that teacher and assessed quality only

Strong factorial invariance was reached for all three dependent variables, which allows for a meaningful interpretation of latent mean change over time (Geiser, 2013). Supplemental Table S2 contains the details of the measurement invariance testing. The model demonstrated acceptable to good model fit: χ^2^(2076, *N* = 663) = 2958.86, *p* < .001, CFI = .90, RMSEA = .025, 95% CI = .023–.027, and SRMR = .047. The percentage of variance explained by the full model was 23.9% for the change in moral disengagement, 15.9% for the change in perceived injunctive class norm, and 43.1% for the change in bullying.

The results of the moderated mediation model indicated that the three-way interaction term was statistically significant at the .10 level for the effect of intervention (X) on change in moral disengagement (*β* = .17, *p* = .06). Probing the interaction showed that intervening on moral disengagement had a significant effect on change in moral disengagement as well as a significant indirect effect on bullying (through moral disengagement) for all the combinations of fidelity and quality levels. Table 2 reports the unstandardized regression coefficient and associated *p*-value for the nine conditional effects on change in moral disengagement and conditional indirect effects on bullying flowing through this mediator.

**Table 2:** Regression coefficient and significance of the conditional effects

	Effect on change in moral disengagement		Indirect effect on bullying	
	β	*p*-value	β	*p*-value
**Combinations**				
Low fidelity–low quality	− 0.42	< .01	− 0.14	.02
Low fidelity–medium quality	− 0.70	< .001	− 0.25	.02
Low fidelity–high quality	− 0.99	< .01	− 0.36	.02
Medium fidelity–low quality	− 0.51	< .01	− 0.16	< .01
Medium fidelity–medium quality	− 0.62	< .001	− 0.22	.01
Medium fidelity–high quality	− 0.74	< .001	− 0.28	.01
High fidelity–low quality	− 0.60	.02	− 0.18	< .01
High fidelity–medium quality	− 0.54	< .001	− 0.19	.01
High fidelity–high quality	− 0.49	< .01	− 0.20	< .01

*N* = 663

Low, medium, and high correspond to the mean (0), one standard deviation below the mean (− 1), and one standard deviation above the mean (+ 1)

**Fig. 4:**
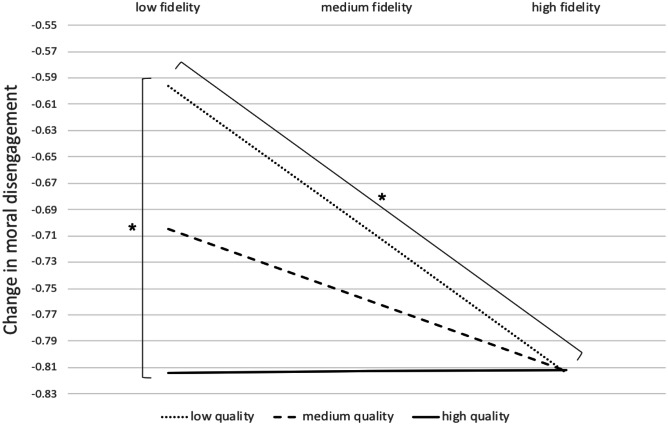
Expected values of change in moral disengagement following the moral disengagement intervention

A closer look at the plot of absolute expected values of change in moral disengagement confirmed that intervening on moral disengagement decreased students’ moral disengagement for all the combinations of fidelity and quality levels. Visual interpretation of Fig. 4 suggests that when fidelity was low, the magnitude of the decrease in moral disengagement was conditional upon the level of quality. Greater quality tended to amplify the reduction. An inferential test indicated that this effect was statistically significant at the .10 level and that an increase of one standard deviation in quality yielded a decrease of *β* =  −.11 in moral disengagement (*p* = .05). As soon as fidelity reached a medium level, however, the effect of quality was no longer significant. On the other hand, the magnitude of the decrease in moral disengagement varied as a function of fidelity when quality was low. An inferential test indicated that this effect was also significant and that an increase of one standard deviation in fidelity yielded a decrease of *β* =  −.11 in moral disengagement (*p* < .01). Again, as soon as quality reached a medium level, the effect of fidelity was no longer significant.

By contrast, there was no significant main or interaction effect of fidelity or quality on the effect of the intervention (X) on change in perceived anti-bullying injunctive class norm.

There was no significant main or interaction effect for the direct effect of intervention (X) on bullying either.

## Discussion

Assessing implementation is critical to interpreting results of anti-bullying interventions (Low et al., 2014). Discerning how implementation fidelity and quality were associated with the outcomes of two promising anti-bullying program components—intervening on moral disengagement and social norms—was the goal of our study. In line with previous research and theory (e.g., Berkel et al., 2011; Durlak & Dupre, 2008; Schulte et al., 2009), we found that both higher fidelity and higher quality were related to greater reduction in moral disengagement, and, indirectly, bullying, for the relevant intervention group. This supports our primary hypothesis that not only fidelity to the intervention plan but also how well teachers deliver the intervention may be important for anti-bullying psychosocial interventions (e.g., Berkel et al., 2011; Schulte et al., 2009).

Our secondary hypothesis that fidelity and quality would interact in influencing the outcomes (Berkel et al., 2011) was also supported for the moral disengagement intervention condition. The association between fidelity and intervention outcomes was conditioned upon quality and vice-versa. When fidelity was low, higher quality was significantly associated with greater outcomes. Thus, teachers’ preparedness, enthusiasm, and other aspects of quality played a critical role when they followed the intervention plan less closely. When the quality of delivery was low, on the other hand, then sticking more closely to the intervention plan was significantly associated with greater outcomes. However, as soon as an average quality level was reached, fidelity no longer mattered. This pattern of results is consistent with findings from other fields. For example, a study on drug counseling found that fidelity was decisive when the therapeutic alliance with the patient was weak, but not when the alliance was strong, as patients typically improved in that case (Barber et al., 2006). In another study, improvement of students’ reading skills was significantly associated with fidelity only when teachers’ instructional quality was below average (Capin et al., 2022).

The various components of high implementation quality may contribute to the establishment of a safe, supportive, and engaging learning environment, thereby enhancing the teacher-student relationship. Within this context, students are more likely to be receptive to interventions from their teachers and better equipped to understand the core educational message, even in cases where teachers deviate from the intended content or procedures. When teachers effectively convey this educational message with high quality, they also implicitly communicate their anti-bullying stance to their students. This communication is likely to influence the social dynamics within the classroom through the influential role of the teacher, often referred to as the “invisible hand” of the teacher (Farmer et al., 2011).

Contrary to our expectations, was the absence of a significant average difference in perceived injunctive class norm change between groups at any combined level of fidelity and quality. In other words, even under ideal implementation conditions, there was no significant average difference in this hypothesized mediator between students who received the relevant intervention or not. This result is important, as it suggests that we can rule out implementation as a potential explanation for the absence of the intended mediation effect. Possible alternative explanations might be a measurement issue (e.g., reporting what they thought to be their classmates’ attitudes might have been too difficult for elementary students), a more relevant reference group, such as the subgroup of friends (Paluck & Sheperd, 2012), the teacher being more influential than the arbitrarily created group of classmates (Veenstra & Lodder, 2022), or the possibility of differential effects of the intervention depending on students’ role in bullying or some (inter)personal characteristics (e.g., popular bullies being more prone to align with the peer norm to protect their social status) (Tolmatcheff et al., 2022a).

Finally, we were curious as to whether the link between the implementation variables and outcomes would differ between the two interventions. Given the absence of an effect on the intended mediator in the social norms intervention condition, we were not able to further test this idea. Nonetheless, we contend that the associations between different implementation features and outcomes are likely to vary across interventions (Durlak & Dupre, 2008; Schulte et al., 2009). For instance, although previous research has roughly estimated that a minimum of 60% fidelity was required for programs to achieve their intended effects (Durlak & Dupre, 2008), it is unlikely that this very cutoff point applies to all interventions, irrespective of their type and nature (Nelson et al., 2012)—especially considering the high heterogeneity of measures and methods used to assess and associate implementation and outcomes (Tolmatcheff, 2021).

### Strengths and Limitations

Strengths of this study are the relatively large student sample with high retention rates, the use of latent change scores, the control for the nested structure of the data, and the use of observational data for assessing fidelity and quality. In addition, the use of a multiplicative moderation model allowed for considerably more flexibility than an additive multiple moderation (Hayes, 2018).

Despite its strengths, this study also has limitations. First, the sample size at the teacher level was relatively small (*N* = 34). Although power analysis indicated that our design has sufficient power to detect significant effects of medium size (see supplemental materials), it is possible that we could not detect a small significant three-way interaction for the perceived injunctive class norm because of a lack of power.

Second, implementation data were collected during one lesson for each teacher, given the available time and human resources for this study. In future studies, we suggest that researchers collect implementation data for each lesson and then average them across the lessons (e.g., Hirschtein et al., 2007) or combine teacher self-reports with observational ratings to ensure criterion validity (e.g., Renshaw & Jimerson, 2012).

Third, although observational data are generally more reliable than self-reports (Durlak & Dupre, 2008; Hirschstein et al., 2007), teachers can be resistant to them (Dusenbury et al., 2003). They may not feel comfortable delivering the intervention in front of raters. Although we were careful to build a relationship of trust and non-judgment with the teachers, being observed while delivering the lesson was nevertheless stressful for some of them, which may have impaired the delivery to some extent.

Finally, a limitation faced by many studies assessing implementation is the difficulty in using data from a control group in the analysis. For instance, it would not have been possible to use a moderation model including the control group in our study, because there was no implementation data for this group. Some studies have created categorical implementation variables (e.g., low, moderate, high) to be able to contrast each level with the control group (e.g., Salmivalli et al., 2005). However, cutoffs used for categorization are always arbitrary and have less statistical power than continuous variables (Durlak & Dupre, 2008). We suggest that using an active control group (in which an intervention of the same duration and intensity but unrelated to bullying is delivered, as in our design) may be an interesting way to solve this issue. Although it is unrelated to bullying, implementation of the control intervention could be assessed as well, thus allowing a relevant comparison in a sophisticated statistical design using continuous moderators. As far as we know, this has never been done before in anti-bullying intervention studies linking implementation to student outcomes, presumably because of the high cost in time and human resources to train and observe teachers in an extra condition.

### Implications and Future Directions

This study highlights that assessing implementation is essential to provide information about the effects of interventions associated with different levels of implementation. Because the implementation of anti-bullying interventions by teachers in natural contexts inevitably fluctuates (Goncy et al., 2015), for a given intervention to be effective even under suboptimal implementation conditions represents a considerable advantage. At least ensuring that the intervention does not produce any iatrogenic effect should be a necessary condition for any intervention to be further disseminated. In addition, clarifying that the limited or null impact of an anti-bullying intervention is not due to variations in its implementation is important in extending our understanding and knowledge of the effective program components and processes involved in bullying.

In terms of cost-effectiveness ratio, our findings demonstrated that the moral disengagement component reduced moral disengagement and had an indirect effect on bullying even when poorly implemented. That student outcomes were significantly impacted by the level of implementation provides further support for the effects being the result of the intervention rather than other factors, such as developmental changes (Haataja et al., 2014). Regarding the social norms component, future studies should test several alternative mediators to clarify which of them mediate change in bullying.

Developing parallel interventions (unrelated to bullying) to be used in an active control group could be a promising direction for evaluations of anti-bullying interventions. Regarding quality, this would enable researchers to distinguish the part of the association with students’ outcomes that is specific to the intervention from the part that is not (i.e., that exists regardless of the delivered content). Although we assessed quality solely in relation to the intervention implementation, our measure likely partially reflects teachers’ personal qualities and broader transversal skills. Conducting further research with active control groups would be beneficial to determine the degree to which these overall individual skills, beyond intervention implementation, are linked to students’ outcomes in relation to bullying.

Our results suggest that the skills reflected in our measure of quality may be particularly desirable from a bullying prevention perspective. As not all teachers are equal when it comes to these pedagogical and relational skills, the finding that higher fidelity seemed to weaken the negative impact of poorer quality can also be seen as a strength. However, our study showed that teachers who had low fidelity scores combined with high-quality scores achieved results as good as those who implemented the intervention with high fidelity. This finding is significant because it challenges the conventional wisdom in the implementation literature that behavior change cannot be achieved through quality alone if the core components of a program are not fully implemented (e.g., Berkel et al., 2011). Moreover, compelling these highly skilled teachers to stick to the intervention could be counter-productive (Quinn & Kim, 2017). This finding also invites us to reconsider teachers’ agentic role in bullying prevention in general, and how we could better support these highly skilled teachers’ agency while providing enough guidance for the less skilled teachers when designing anti-bullying interventions.

To conclude, we examined the association between implementation fidelity and quality and the effects of two different anti-bullying components. For the moral disengagement component, high quality—which reflected various pedagogical and relational aspects of the delivery—appeared to be beneficial in any case and essential when teachers implemented the intervention less rigorously. However, when teachers lacked these skills, then fidelity mattered. The social norms component had no effect on the hypothesized mediator regardless of the fidelity and quality levels, thus ruling out implementation as a possible explanation for the absence of this effect. We advocate the systematic assessment of implementation in evaluation studies to expand our understanding of how anti-bullying programs work and knowledge of their effective components.

### Supplementary Information

Below is the link to the electronic supplementary material.

Supplementary file1 (DOCX 236 KB)
