# Supplementary material for: Examining the Link Between Implementation Fidelity, Quality, and Effectiveness of Teacher-Delivered Anti-Bullying Interventions in a Randomized Controlled Trial
Source: Prev Sci. 2023 Sep 1;25(3):407–20. doi: 10.1007/s11121-023-01580-8 (PMC11093827; doi:10.1007/s11121-023-01580-8)
Supplement: Supplementary file 1 — Supplementary file1 (DOCX 236 KB) [file 11121_2023_1580_MOESM1_ESM.docx]

**Anti-Bullying Interventions Delivered by Teachers: Examination of the Influence of Implementation Fidelity and Quality in a Randomized Controlled Trial**

**Supplemental materials**

***Prevention Science***

Chloé Tolmatcheff ^1, 2^, René Veenstra^1^, Isabelle Roskam^2^, and Benoit Galand^2^

^1^ Department of Sociology, University of Groningen

^2^ Psychological Sciences Research Institute, University of Louvain

**Author Note**

Chloé Tolmatcheff
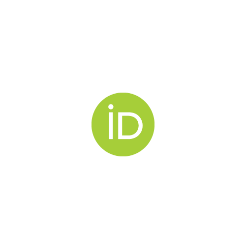
 https://orcid.org/0000-0002-5833-9176

René Veenstra
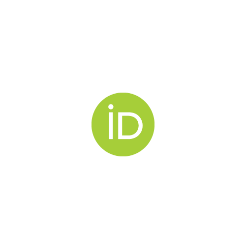
 https://orcid.org/0000-0001-6686-6307

Isabelle Roskam
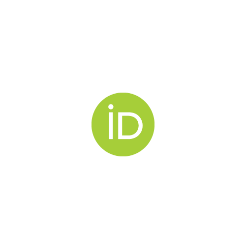
 https://orcid.org/ 0000-0002-1449-1133

Benoît Galand
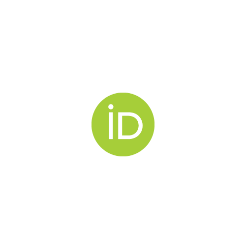
 https://orcid.org/0000-0003-3387-4305

Correspondence concerning this article should be addressed to Chloé Tolmatcheff, Department of Sociology, University of Groningen, Grote Rozenstraat 31,

9712 TG Groningen, the Netherlands. Email: chloe.tolmatcheff@gmail.com

**Supplemental materials**

Preliminary note: The following online supplemental materials partially overlap with the content included as supplemental materials of “The effectiveness of moral disengagement and social norms as anti-bullying components: A randomized controlled trial”, by Tolmatcheff et al., 2022a, *Child Development, 93*, https://doi.org/10.1111/cdev.13828.

**Power Analysis**

Power analysis, conducted in Optimal Design (Raudenbush et al., 2011), indicated that a two-level cluster RCT design with 34 clusters and ICCs of .030 (moral disengagement) and .037 (perceived injunctive class norm) at the class level leads to a power of .80 to detect an effect size of approximately .25 for a significance level of α =.10. For the original sample size determination (including the control group), please see Tolmatcheff et al., 2022a.

*
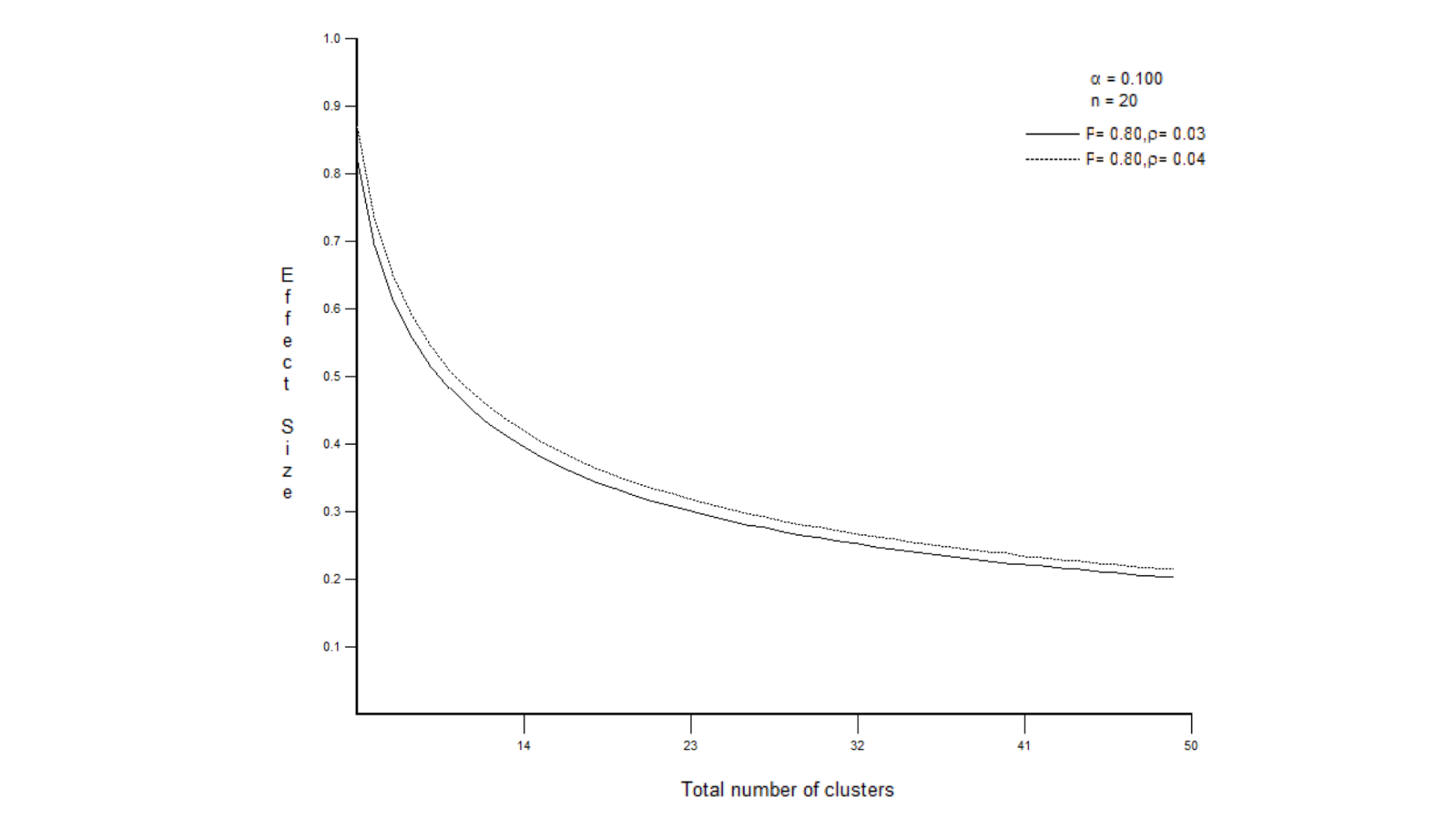
*

*Note.* We set the significance level to α = 0.10 because the number of clusters was relatively small (*J*=34), and we might lack statistical power to test the relevant interaction effects in our study. We set the cluster size (*n*) to 20 as it is the average number of students per classroom in elementary schools in French-speaking Belgium (OECD, 2013).

**School Recruitment and Eligibility Criteria**

Schools were recruited on a voluntary basis. The recruitment drive was launched in September 2018 and was broadcast among schools through various contacts in the field. A letter describing the research project presented both the costs (the number of hours required for data collection, the training session, and the intervention) and the benefits that schools could expect. Each school would receive a detailed assessment of their bullying situation and a day of anti-bullying training for the whole teaching team for free. In addition, in the two experimental conditions, Grades 4-6 teachers would also receive a specific training day and personalized supervision throughout the implementation of the intervention for free. In total, 25 schools expressed interest in the project. These schools were contacted by phone by the first author to give them an outline of the project. Out of the 25 schools, three did not match the eligibility criteria (they had a preschool or Grades 1-3 section only, or less than five teachers in Grades 4-6), seven said they lacked time and/or had too many concurrent projects, two were hesitant, and four did not follow up after the phone contact. Nine school principals confirmed their interest immediately during the call. The first author met the Grades 4-6 teachers of these schools between mid-October and mid-November to present the collaboration project. Teachers were informed about the randomization process. At the end of the meeting, they filled in a short confidential form to indicate whether they were willing to join the project. A minimum of five teachers per school was required to seal the agreement. All agreed to participate in the project (*N* = 57).

**Demographic Characteristics**

These schools differed in school size, ranging from 94 to 213 students in Grades 4-6. They were also diverse in terms of geographical location — covering four out of the six provinces of the French-speaking part of Belgium. Finally, they were diverse in terms of socioeconomic level: their official socioeconomic indices varied from 1 to 19. A school’s socioeconomic index (ISE) is an official figure calculated annually and ranging from 1 to 20, based on five socioeconomic indices of the students’ area of residence — per capita income, level of education, unemployment rate, professional activities, and housing comfort. Each school was then randomly assigned to one of the three conditions by the first author using a draw procedure. To avoid inequitable group sizes, we randomly assigned the three biggest schools to each of the three conditions. Table S1 provides demographic characteristics of the three conditions.

| **Table S1** | | | |
| --- | --- | --- | --- |
| *Demographic Information at T1* | | | |
|  | Intervention on moral disengagement | Intervention on class norms | Control group |
| Boys (%) at T1 | 49.7% | 49.1% | 44.3% |
| Age *M* (*SD*) at T1 | 10.13 (1.06) | 10.28 (0.98) | 10.05 (1.02) |
| Grades |  |  |  |
| 4 | 38.1% | 29.1% | 35.7% |
| 5 | 31.8% | 24.1% | 30.2% |
| 6 | 30.1% | 46.9% | 34.1% |
| Geographical location (Province) | Liège^1^  Brussels^6^  Namur^8^ | Brussels^2^  Namur^3^  Namur^4^ | Brabant wallon^5^  Brussels^7^  Brabant wallon^9^ |
| School size (number of students in Grades 4-6) | 98^1^  168^6^  108^8^ | 129^2^  131^3^  137^4^ | 142^5^  213^7^  94^9^ |
| School ISE | 7^1^  1^6^  10^8^ | 9^2^  12^3^  19^4^ | 14^5^  1^7^  15^9^ |
| *Note.* Schools are indicated by superscript numbers (e.g., school number 1 was located in the Province of Liège, had 98 Grades 4-6 students, and had a socioeconomic index of seven).  Although there is no census of students’ ethnic background in this part of Belgium, students from a non-Belgian ethnic background are especially present in the Region of Brussels, corresponding to schools #2, #6, and #7 (Sacco et al., 2016).  T1 = Time 1 | | | |

**Specific Culturally-Related Constraints in the Data Collection**

In the French-speaking part of Belgium, schools do not register confidential demographic information such as the socioeconomic status of students’ families. They only have access to the school’s average socioeconomic index (ISE), as described in the previous section. Another culturally-related constraint in the demographic data collection relates to students’ ethnic background: asking students about their race or ethnicity would be considered inappropriate, especially in elementary schools. As an illustration, there is no official census of students’ ethnic background beyond their nationality in the (French-speaking part of Belgium) (Jacobs & Rea, 2015). However, researchers regularly indicate that students from non-Belgian ethnic backgrounds are present in particularly large numbers in the Region of Brussels (Sacco et al., 2016).

**Timeline**

Teachers from Grades 4 to 6 attended the training day between mid-January and the end of February 2019. Schools began the intervention within a month after the training. The pre-test data collection (T1) took place between mid-November and mid-December 2018. The post-test data collection (T2) took place between the very end of March and mid-May. This somewhat longer period for the second data collection was because of the two-week spring break. On average, there was a time lapse of 15 weeks between the two waves. Schedule variations between schools were due to school vacations, school trips, and other appointments. For instance, in some schools, Grade 6 students went on a two-week skiing trip with their class teachers after Christmas break. However, the time lapse between the end of the intervention and the second wave of data collection was the same for all schools (1-2 weeks).


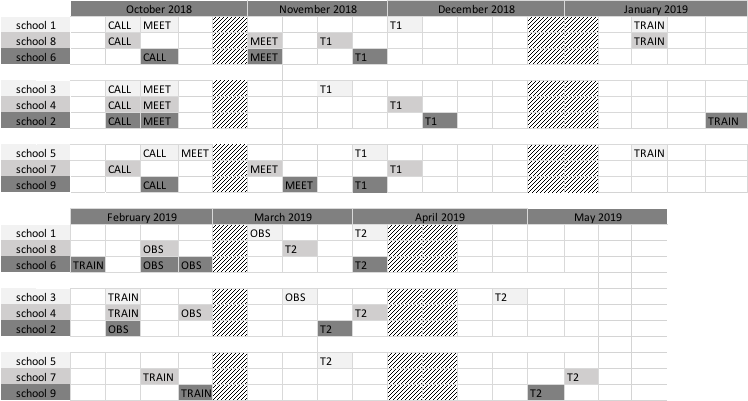


*Note.* Columns represent weeks. Cross-hatching represents school vacations. CALL = first (phone) contact with the schools' principals; MEET = first meeting with the Grades 4–6 teachers to present the project; T1 = first wave of data collection; TRAIN = training day for Grades 4–6 teachers; OBS = implementation data collection day, corresponding to lesson 3 for the first intervention (schools 1, 8, 6) and lesson 2 for the second intervention (schools 3, 4, 2); T2 = second wave of data collection.

**Data Collection**

Parental consent had been asked prior to data collection through passive consent forms sent by the schools. Students did not participate if parents objected to their participation or if they themselves did not want to participate. Students completed confidential paper questionnaires during class hours under the supervision of one to two research team members, who were available to answer questions. In a five-minute oral introduction, the researchers presented the goal of the survey (“investigating children’s well-being at school”) and a few basic rules. Students were reminded that the questionnaire was strictly confidential and not mandatory. For follow-up purposes, they were asked to create an identification code by joining the first two letters of their first name and the first two letters of their surname to the number of the day of their birthday (e.g., MA+DU+14). In Grade 4 classes (including two multi-grade classes with students from both Grades 3 and 4), the questionnaire was read aloud by a researcher to compensate for possible reading difficulties of students of this age. Non-response rates due to students’ absences were higher at T1 than at T2 because we left empty questionnaires with pre-stamped and pre-addressed envelopes with the teachers at T2. This way, students who had missed the data collection for some reason could participate within the following two weeks. Implementation data were collected by two raters (the principal researcher and a trained master’s student) who both attended the same lesson in each intervention classroom. As all lessons in the first intervention unfolded similarly and contained the key intervention elements (invalidating the moral disengagement mechanisms), we selected the one in the middle (lesson 3) for observation. For the second intervention, we observed lesson 2 as it included both the key message about the norm misperception and the creation of the norm-based messages, which are key elements of the intervention. The whole procedure was approved by the Psychological Sciences Research Institute Ethics Committee of the University of Louvain. See also the CONSORT Checklist below.

**Teacher Training**

The teachers’ training day in the two intervention groups was divided into two parts: the first part was devoted to bullying in general and the second part to the specific intervention (moral disengagement or social norms, depending on the group). The part on bullying aimed to provide general knowledge and to raise awareness about the subject among teachers. Teachers were sensitized to characteristics, forms, participants’ roles, social dynamics, prevalence, and consequences of bullying. The second part of the training started with a quick presentation of the theoretical and empirical foundations of the intervention. Next, teachers were instructed how to use the material. Then, they were immersed in an accelerated simulation of the five intervention sessions to familiarize them with the content and enable them to gain a feel for the expected mechanisms of change for themselves. Finally, various challenging situations that could occur during the sessions were envisaged (e.g., What if a student shows resistance to or makes fun of the proposed activities?) to discuss the most appropriate reactions and exchange tips. Close monitoring was provided during the intervention period by means of exchanges in newly created WhatsApp or Facebook groups.

The teachers’ training day in the control group started with a reminder of the whole research project’s design and of the importance of the control group for the evaluation process. Then, the main issues relating to the world environmental crisis were presented to highlight the purpose of delivering this intervention to their students. Next, teachers were given an explanation of how to use the material; the sessions were described and the teachers were immersed in an accelerated simulation of the five intervention sessions. Close monitoring was also provided for teachers of the control group over the weeks of intervention.

**Intervention 1: Moral Disengagement Component**

The first intervention used in our study is inspired by the Bullying Literature Project-Moral Disengagement Version (hereafter BLP-MD; Wang & Goldberg, 2017), which is a five-session class-wide intervention that uses storybooks displaying bullying situations to prevent bullying among elementary children. It includes story reading, discussions with students, writing activities, and role play. A pilot study of the BLP-MD revealed a significant decrease in both moral disengagement and victimization in intervention classes in 7-9 years old elementary students (Grade 3) (Wang & Goldberg, 2017). We did not use the BLP-MD storybooks in our study for two reasons. First, we thought they would be perceived as “too childish” by the 9-12 years old students in our sample (Grade 4-6). Second, we judged that the bullies portrayed within these stories were rather stereotypical and presented as “the big mean bully that everyone is afraid of.” This simplistic view of bullying does not reflect the increasingly recognized diversity of profiles among and motives of bullies (e.g., Garandeau, 2021; Tolmatcheff et al., 2018). We therefore created new stories of around 1,800 words each, which were adapted to the age of the students in our sample and designed to represent various bullying situations (e.g., same-sex or cross-sex bullying, provocative or non-provocative victims, traditional or cyber bullying, bullying extended to the whole class or restricted to a few students, prejudice-related bullying, bullying stemming from some conflict). The stories were intended to illustrate the use of moral disengagement mechanisms by the bullies. They did not contain other anti-bullying components such as bystanders’ strategies for supporting the victim, because our goal was to assess the effects of intervening on moral disengagement only. As such, the stories did not describe a resolution of the situation for the victims at the end. Instead, students were invited to identify the moral disengagement mechanisms (presented as “little lies” on which the bullies relied to avoid guilt), which were then invalidated by the teachers. A typical session unfolded as follows: First, the teacher read the story aloud before handing out the written version of the story. Then, students were invited to answer individual questions (inserted in the written story) to reflect on the situation (e.g., “When [name of the bully] says that ‘it was just a joke’, do you think it is true?”). The teacher then chose one or two volunteers to share their answers with the others. Next, the teacher invited students to identify collectively the mechanism(s) used by the bullies in the story. Once identified, students were invited to take a minute of individual reflection to think about their own experience of using these mechanism(s). Then, the teacher reminded the students that moral disengagement mechanisms are false excuses that do not make the harmful behavior more acceptable. Students were finally invited to write the (simplified) name of the mechanism(s) discovered during the session on an individual summary sheet. Just like in the original program, every session was closed with an individual writing activity (e.g., writing the end of the story, making a bookmark to illustrate the mechanisms, committing to stop using the moral disengagement mechanisms in a confidential letter to themselves that would only be reopened by the students at the end of the school year).

Our research design implied to assess anti-bullying components separately. For that reason, we focused only on students’ use of moral disengagement mechanisms. In contrast to the BLP-MD, we did not use role play, which can enhance empathy (Bagès et al., 2021). Thus, we tried as much as possible not to manipulate any other component.

The English translation of the stories can be found at the end of the supplemental materials. An example of a story summary illustrating euphemistic labeling and advantageous comparison is: “At the beginning of the school year, Lumi finds out her best friend is no longer in her class. Two girls in her class start to whisper and laugh every time she answers the teacher’s questions – Lumi has no idea why. During a group work session, the two girls reject Lumi and make fun of her. When the teacher notices, the two bullies claim that ‘it was just a joke’ and that ‘it is not that bad compared to insulting her.’ They prevent Lumi from making new friends and mock a classmate who tries to defend her. One day, Lumi ends up running out of the classroom, sobbing.”

**Intervention 2: Social Norms Component**

The second intervention used in our study is based on the “Survey of Bullying at Your School” project, which is a school-wide intervention using students’ online self-reported data to highlight the discrepancy between perceived and actual pro-bullying norms. A pilot study of the project in 11-14 years old students (Grade 6-8) revealed a significant decrease in both perceived pro-bullying injunctive norms and bullying and victimization (Perkins et al., 2011). In the original project, social norm messages about bullying were created by the research team based on the online pre-test data. These messages were displayed on posters in the schools to provide students with feedback about the actual norms, which were widely misperceived. We modified this procedure to let students discover their actual injunctive class norms toward bullying by themselves, in a more entertaining, instant, and offline format, better suited to our participants’ age. During the first session, each student received ten small sheets with yes/no questions. The sheets referred to five very common bullying behaviors — bugging, excluding, making fun, insulting, and pushing/hitting. Five questions related to the perceived pro-bullying injunctive class norm and five other questions related to the corresponding personal pro-bullying attitude. In other words, the same behavior (e.g., excluding someone) was evaluated by each student in terms both of the perceived classmates’ attitudes (e.g., “Most of my classmates think that excluding someone is OK” – yes/no) and personal attitude (e.g., “I think that excluding someone is OK” – yes/no). After the questions had been answered anonymously, the sheets were gathered in, then divided by type of behavior. Students were split into five work groups that received the sheets for both perceived injunctive class norm and personal attitude related to one of the behaviors. The composition of the groups had been decided on carefully based on the pre-test data and in collaboration with the class teachers prior to the intervention. Suspected bullies were mixed with prosocial students and key roles in the group were attributed to well liked students who were not involved in bullying. Each group was asked to count the number of yes and no answers related to the perceived norm and the personal attitude. The final counts of each group were displayed in colors on two parallel posters to visualize the discrepancy between students’ perceived attitudinal class norm and their actual class norm. For instance, the perceived norm poster might show that 12 out of 20 students thought that most classmates regarded excluding someone as OK, whereas the actual norm poster would show that only two out of 20 students thought it was OK. This discrepancy was underlined by the teacher and the *actual* norms of the class were emphasized. In the following sessions, groups were invited to create by themselves a slogan to describe their class’s actual norm (e.g., “In our classroom, we want to accept everyone”). The slogans were supposed to emphasize the desired prosocial behavior rather than the condemned antisocial behavior (e.g., “We accept everyone” rather than “We do not reject anyone”). Eventually, groups were invited to make an artistic poster with the slogan for display in the school’s corridors. In the final session of the intervention, each class presented the posters to students of another class, and vice-versa.

| **Table S2**  *Testing for Factorial Invariance Across Time* | | | | | | | | | | | |
| --- | --- | --- | --- | --- | --- | --- | --- | --- | --- | --- | --- |
|  | | | | 90% CI for RMSEA | |  | | | | | |
| Model | 𝜒^2^ | df | RMSEA | LL | UL | ∆  RMSEA | CFI | ∆  CFI | TLI | SRMR | ∆  SRMR |
| **Moral disengagement** |  |  |  |  |  |  |  |  |  |  |  |
| M0 configural model | 657.5*** | 321 | .030 | .027 | .033 | _ | .933 | _ | .922 | .037 | _ |
| M1 weak invariance 1^st^ | 667.9*** | 328 | .030 | .027 | .033 | 0 | .933 | 0 | .922 | .038 | .001 |
| M2 weak invariance 2^nd^ | 679.7*** | 334 | .030 | .027 | .033 | 0 | .932 | -.001 | .923 | .039 | .001 |
| M3 strong invariance 1^st^ | 700.2*** | 341 | .030 | .027 | .033 | 0 | .929 | -.003 | .921 | .039 | 0 |
| M4 strong invariance 2^nd^ | 731.8*** | 347 | .031 | .028 | .034 | .001 | .924 | -.005 | .917 | .040 | .001 |
| *N* = 1163 | | | | | | | | | | | |
| **Perceived injunctive class norm** |  |  |  |  |  |  |  |  |  |  |  |
| M0 configural model | 46.4 | 38 | .014 | 0 | .026 | _ | .996 | _ | .992 | .018 | _ |
| M1 weak invariance | 51.4 | 46 | .010 | 0 | .023 | -.004 | .997 | .001 | .996 | .021 | .003 |
| M2 strong invariance | 63.6* | 50 | .015 | 0 | .026 | .005 | .993 | -.004 | .990 | .023 | .002 |
| *N* = 1161 | | | | | | | | | | | |
| **Bullying** |  |  |  |  |  |  |  |  |  |  |  |
| M0 configural model | 199.9** | 145 | .018 | .011 | .024 | _ | .979 | _ | .973 | .042 | _ |
| M1 weak invariance | 191.9* | 158 | .014 | .004 | .020 | -.007 | .987 | .008 | .985 | .035 | -.007 |
| M2 strong invariance | 205.5* | 165 | .015 | .006 | .021 | .001 | .985 | -.002 | .982 | .036 | .001 |
| *N* = 1163  1^st^ = first-order, 2^nd^ = second-order  * p < .05. ** p < .01. *** p < .001. | | | | | | | | | | | |

**Distribution of Fidelity and Quality in each Intervention Condition**

*Note*. Dots of same color belong to the same school.

*Note*. Dots of same color belong to the same school.

**Additional analysis**

An additional analysis revealed that intervening on moral disengagement had a significant indirect effect on indirect and direct (but not cyber) bullying through change in moral disengagement for all the combinations of fidelity and quality levels. Table S3 presents the regression coefficient and associated *p*-value for the nine conditional indirect effects on indirect, direct, and cyber bullying flowing through change in moral disengagement.

| **Table S3**  *Regression Coefficient and Significance of the Conditional Indirect Effects* | | | | | | | | | | | |
| --- | --- | --- | --- | --- | --- | --- | --- | --- | --- | --- | --- |
|  | Indirect bullying | | |  | Direct bullying | | |  | Cyber bullying | | |
| **Combinations** | *β* |  | *p*-value |  | *β* |  | *p*-value |  | *β* |  | *p*-value |
| low fidelity – low quality | –0.13 |  | .02 |  | –0.09 |  | .03 |  | –0.08 |  | .23 |
| low fidelity – medium quality | –0.21 |  | .02 |  | –0.16 |  | .03 |  | –0.14 |  | .23 |
| low fidelity – high quality | –0.30 |  | .02 |  | –0.22 |  | .04 |  | –0.20 |  | .23 |
| medium fidelity – low quality | –0.15 |  | .01 |  | –0.11 |  | .03 |  | –0.10 |  | .23 |
| medium fidelity – medium quality | –0.19 |  | < .01 |  | –0.14 |  | .02 |  | –0.12 |  | .22 |
| medium fidelity – high quality | –0.22 |  | < .01 |  | –0.16 |  | .02 |  | –0.15 |  | .23 |
| high fidelity – low quality | –0.18 |  | .04 |  | –0.13 |  | .07 |  | –0.12 |  | .26 |
| high fidelity – medium quality | –0.17 |  | < .01 |  | –0.12 |  | .03 |  | –0.11 |  | .24 |
| high fidelity – high quality | –0.15 |  | .02 |  | –0.11 |  | .05 |  | –0.10 |  | .27 |
| *N=*663  *Note.* Low, medium, and high correspond to the mean (0), one standard deviation below the mean (-1), and one standard deviation above the mean (+1). | | | | | | | | | | | |


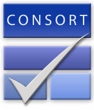
CONSORT 2010 checklist of information to include when reporting a randomized trial

| Section/Topic | Item No | Checklist item | Reported on page No |
| --- | --- | --- | --- |
| Title and abstract | | | |
|  | 1a | Identification as a randomized trial in the title | ☑ |
|  | 1b | Structured summary of trial design, methods, results, and conclusions (for specific guidance see CONSORT for abstracts) | ☑︎ |
| Introduction | | | |
| Background and objectives | 2a | Scientific background and explanation of rationale | ☑︎See introduction, specifically *Influence of implementation in anti-bullying programs* and *Moral Disengagement and Social Norms as Targets of Anti-Bullying Program Components* |
|  | 2b | Specific objectives or hypotheses | ☑︎See *The present study* section |
| Methods | | | |
| Trial design | 3a | Description of trial design (such as parallel, factorial) including allocation ratio | ☑︎See *Sampling and Design* and *Figure 1* (*Flowchart of recruitment and retention*), and *Supplemental materials* |
|  | 3b | Important changes to methods after trial commencement (such as eligibility criteria), with reasons | Not applicable |
| Participants | 4a | Eligibility criteria for participants | ☑︎See *Supplemental materials* |
|  | 4b | Settings and locations where the data were collected | ☑︎See *Supplemental materials* |
| Interventions | 5 | The interventions for each group with sufficient details to allow replication, including how and when they were actually administered | ☑︎See *Supplemental materials* |
| Outcomes | 6a | Completely defined pre-specified primary and secondary outcome measures, including how and when they were assessed | ☑︎See *The Present Study,* *Data collection and Participants,* and *Measures* |
|  | 6b | Any changes to trial outcomes after the trial commenced, with reasons | Not applicable |
| Sample size | 7a | How sample size was determined | ☑︎See *Sampling and Design* and *Supplemental material* |
|  | 7b | When applicable, explanation of any interim analyses and stopping guidelines | Not applicable |
| Randomization: |  |  |  |
| Sequence generation | 8a | Method used to generate the random allocation sequence | ☑︎See *Supplemental materials* |
|  | 8b | Type of randomization; details of any restriction (such as blocking and block size) | ☑︎See *Supplemental materials* |
| Allocation concealment mechanism | 9 | Mechanism used to implement the random allocation sequence (such as sequentially numbered containers), describing any steps taken to conceal the sequence until interventions were assigned | ☑︎See *Supplemental materials* |
| Implementation | 10 | Who generated the random allocation sequence, who enrolled participants, and who assigned participants to interventions | ☑︎See *Supplemental materials* |
| Blinding | 11a | If done, who was blinded after assignment to interventions (for example, participants, care providers, those assessing outcomes) and how | ☑︎Blinding was not applied. Schools, teachers, and researchers were aware of the intervention or control status of schools. |
|  | 11b | If relevant, description of the similarity of interventions | ☑︎See *Teacher training and interventions* in the Introduction *and Supplemental materials* |
| Statistical methods | 12a | Statistical methods used to compare groups for primary and secondary outcomes | ☑︎See *Analytical Strategy* |
|  | 12b | Methods for additional analyses, such as subgroup analyses and adjusted analyses | ☑︎See *Analytical Strategy* and *Supplemental materials* |
| Results | | | |
| Participant flow (a diagram is strongly recommended) | 13a | For each group, the numbers of participants who were randomly assigned, received intended treatment, and were analyzed for the primary outcome | ☑︎See *Figure 1* (flowchart) |
|  | 13b | For each group, losses and exclusions after randomization, together with reasons | ☑︎See *Figure 1* (flowchart) |
| Recruitment | 14a | Dates defining the periods of recruitment and follow-up | ☑︎See *Supplemental materials* |
|  | 14b | Why the trial ended or was stopped | Not applicable |
| Baseline data | 15 | A table showing baseline demographic and clinical characteristics for each group | ☑︎See *Table S1 (Supplemental materials*) for gender, age, grade, geographical location, school’s size and socioeconomic level |
| Numbers analyzed | 16 | For each group, number of participants (denominator) included in each analysis and whether the analysis was by original assigned groups | ☑︎See *Figure 1* (flowchart) |
| Outcomes and estimation | 17a | For each primary and secondary outcome, results for each group, and the estimated effect size and its precision (such as 95% confidence interval) | ☑︎See *Table 2*. |
|  | 17b | For binary outcomes, presentation of both absolute and relative effect sizes is recommended | Not applicable |
| Ancillary analyses | 18 | Results of any other analyses performed, including subgroup analyses and adjusted analyses, distinguishing pre-specified from exploratory | ☑︎See *Supplemental materials* |
| Harms | 19 | All important harms or unintended effects in each group (for specific guidance see CONSORT for harms) | ☑︎See *Results* |
| Discussion | | | |
| Limitations | 20 | Trial limitations, addressing sources of potential bias, imprecision, and, if relevant, multiplicity of analyses | ☑︎See *Discussion (Strengths and Limitations)* |
| Generalizability | 21 | Generalizability (external validity, applicability) of the trial findings | ☑︎See *Discussion (Strengths and Limitations)* |
| Interpretation | 22 | Interpretation consistent with results, balancing benefits and harms, and considering other relevant evidence | ☑︎See *Discussion* |
| Other information | | |  |
| Registration | 23 | Registration number and name of trial registry | Not available |
| Protocol | 24 | Where the full trial protocol can be accessed, if available | Not available |
| Funding | 25 | Sources of funding and other support (such as supply of drugs), role of funders | ☑︎ |

Moral disengagement intervention: Stories

Preliminary note: Because we wanted to avoid that there be students having the same name as the stories’ characters, we mainly used made-up or rare first names in the original version of the stories (in French). For the present translation, however, we replaced the original names by common first names in English. The grade of the main character was adapted for each classroom (e.g., Grade 5 if the story was read to Grade 5 students).

**Story 1**

Since she was a little girl, **Barbara** has been going to the school in her neighborhood, just a few blocks from her home. This year she is entering Grade 4/5/6. Last year, her best friend **Mary** was in the same class as her. Unfortunately, during the summer, her parents had to move to the other side of the country because of their work, so **Mary** changed schools... **Barbara** cried a lot when she said goodbye to her on the last day of school.

So, on the first day of school, **Barbara** is a little stressed. In class, she meets her new teacher, Mrs. **Johnson**, who seems quite nice. Somewhat reassured, **Barbara** has a look at the other children in the class. There are a lot of new kids. Five of the boys were already in her class last year, but she doesn’t know any of the girls. Mrs. **Johnson** starts by checking that they haven’t forgotten everything during the summer and asks them some math questions. No problem, **Barbara** loves math! She knows all the answers and puts her hand up to answer all Mrs. **Johnson**’s questions. "My goodness, you're really good at math!" exclaims the teacher. **Barbara** blushes proudly at this compliment, but at that moment she notices one of the girls leaning over to her neighbor and whispering something in her ear while staring at **Barbara.** Slightly surprised, **Barbara** answers Mrs. **Johnson**'s next question and then glances at the two girls. Again, the same thing happens: the first girl whispers in the other's ear, and this time they both laugh while looking at **Barbara**. **Barbara** feels very uncomfortable, and doesn't dare put up her hand to answer Mrs. **Johnson**'s questions anymore.

At recess, still a little disturbed by what happened in class, **Barbara** wonders who she'll play with now that **Mary** is gone. Suddenly, **Barbara** notices that three girls from her class are sitting on a bench about ten meters away and are looking at her and laughing. Among them, **Barbara** recognizes the two girls who were whispering in class before recess. Very embarrassed, **Barbara** decides to walk away and heads for the bathroom. When she passes the bench where the three girls in her class are sitting, they burst out laughing. **Barbara** pretends not to notice, but she's sure she's turned as red as a tomato. When she arrives, the bathroom is empty. **Barbara** decides to stay there until the end of recess.

Once back in class, **Barbara** tries to avoid notice and doesn't put her hand up once when Mrs. **Johnson** asks questions, for fear of making the other girls laugh again. Meanwhile, she learns their names. The tallest one, the one who was whispering in class, is **Donna**. Her neighbor is **Grace**, and the one who joined them on the bench in the schoolyard is **Talisa**. The rest of the day passes without incident, but **Barbara** feels very depressed when her mom comes to pick her up after school.

The next day, when she gets to school, **Barbara** feels very lonely. She has tears in her eyes as she thinks about all the good times she had with Mary last year. She decides to hide in the bathroom so no one will see her. Opening the door, she finds herself face to face with **Talisa**, one of the girls in her class who was laughing on the bench in the schoolyard the day before. Quickly, **Barbara** runs into one of the cubicles so that **Talisa** doesn't see that she's crying. When the bell rings, **Barbara** leaves the bathroom, her eyes still red. As **Barbara** walks back to class, she hears **Talisa** say to **Donna** and **Grace** behind her, "I swear, she *was crying*!".

Back in the classroom, Mrs. **Johnson** announces that the next activity will be in groups and starts to make up the groups: "James, you go with Laure and Jean. Robert, with **Donna** and Michael. **Talisa,** with **Grace** and **Barbara...**". **Barbara** hears **Grace** say to **Talisa** "Oh no, not her...".

The children gather in their groups in the classroom. **Barbara** approaches **Grace** and **Talisa** with her head down. There is a third chair next to them, but **Grace**'s schoolbag is on it. As she passes them, **Donna** exclaims, "Are you girls with the cry-baby? Bad luck! **Grace** and **Talisa** laugh, and **Barbara**, who is still standing, looks at her feet without saying anything. "**Grace**, take your schoolbag off there! You can see that **Barbara** can't sit down!", says Mrs. **Johnson**. **Grace** sighs and rolls her eyes, and with exaggeratedly slow gestures removes her schoolbag from the chair. **Barbara,** who would like someone to wave a magic wand and make her disappear right now, sits down slowly.

During the activity, **Barbara** doesn't dare to say anything and remains silent. **Grace** and **Talisa** don't speak to her and do everything together, ignoring her presence. At the end of the activity, each child returns to his or her seat. As **Grace** gets up, she exclaims, "**Barbara** didn't do anything, she left us to do everything by ourselves!". "Not only is she a cry-baby, she's also selfish!", **Donna** shouts from the other end of the room, which makes the other students laugh. Mrs. **Johnson** comes up to her and says, "That's not nice what you just said, **Donna**. I want you to apologize." With a big smile, **Donna** answers, "But I didn't mean it, it was only a joke! We were just having a laugh!".

As **Barbara** leaves the classroom to go to recess, she hears **Talisa** say to **Donna**, "Did you see? She told you off because of **Barbara**!". "That's right, it's so unfair", says **Grace**. "It's not like you insulted her either!", she adds.

When she wakes up the next morning, **Barbara** doesn’t feel well: her stomach hurts. "Mommy, I think I'm sick, I can't go to school." Her mom is a little surprised, because **Barbara** is never usually sick. She put her hand on her daughter's forehead: "No, honey, you don't even have a fever. Come on, get dressed before we're late!"

Mrs. **Johnson** is late that morning. The students go into class to wait for her. **Barbara** walks up to a girl she has never spoken to before, **Charlotte**. "Hello", **Barbara** ventures. "Hello", **Charlotte** replies. **Donna**, who has arrived behind **Barbara'**s back, exclaims, "Yuck! You're talking to the cry-baby? Look out, or she'll start following you and sniveling all the time!" **Grace** and **Talisa** are behind her, laughing. **Charlotte** hesitates, then looks with disgust at **Barbara** and answers, "No, I don't talk to cry-babies" as she walks away.

"You're really mean to her," comments **Andrew**, one of the boys in the class, who was watching. "It's fine, we're just teasing her a little, it's nothing!", answers **Donna**. "Are you in love with her or something?" **Donna** and **Talisa** burst out laughing. "Whatever, I don't care about her!", answers **Andrew** and walks away from the guffawing girls. **Barbara**, who has been silent during the whole exchange, can no longer hold back her tears, which begin to flow down her cheeks. "Ha ha ha! The cry-baby is sad because she's lost her boyfriend!", exclaims **Donna**, to the delight of the two other girls. Other students in the class around her also start laughing and chanting, "Cry-baby! Cry-baby!". **Barbara**, in tears, finally runs out of the classroom. **Donna**, **Grace** and **Talisa** look at each other, feeling slightly unsure of themselves. "We were only teasing her", says **Donna**. The other two girls nod. "Honestly, it's not like we hit her or something!", adds **Talisa**. "Yeah, if we hit her, that would be really mean", says **Grace**. Feeling reassured, the three girls sit quietly in their places waiting for Mrs. **Johnson**.

**Story 2**

Last year, **Ethan** was in a different school and things didn't go well at all. Two boys in his class had chosen him as their victim at the beginning of the school year and they kept on bugging him all year long. But **Ethan** isn't the kind of boy who lets himself be picked on: he's quite tall and actually quite strong. But the other two boys were the toughest in the school: all the students were afraid of them and nobody dared to stand in their way. Toward the end of the year, they even started to bully **Ethan** into giving them his snack, and later his pocket money. When **Ethan**'s parents heard about this, they offered to move him to another school the following year. **Ethan** agreed, and has been determined from the start not to let anyone push him around. Today, **Ethan** has been in his new school for a week, in Grade 4/5/6. He is very happy because he managed to make two friends in his class from the first day: **Duante** and **Daniel**. The three boys get along brilliantly. **Daniel** has already told **Ethan** that he will invite him to his birthday party next month. And **Duante** has asked the other two to spend Saturday afternoon at his house to play with the PS4 he was given during the summer.

**Ethan** also likes his teacher, Mr. **Brown**, very much: he is really nice and comes up with activities for his students that are great fun. For example, in two weeks, they're going to visit the dinosaur museum, and at the end of the day, they're going trampolining. The other students in the class also seem pretty cool: **Ethan** gets along quite well with everyone and even finds the girls nice. In fact, there is only one student in the class that **Ethan** doesn't like: **William**. And he's not the only one: everyone finds **William** a little strange, a little different. He has no friends in the class. Even physically, **William** is different: he's very pale, terribly thin and much smaller than the other students in the class. And also, every time he wants to say something, he can't help speaking very loudly, almost shouting. As a result, he's a bit deafening and the other students avoid him because of this. **Ethan** is sure that even Mr. **Brown** is annoyed by **William**, and it shows: He doesn't speak to him as nicely as to the other students.

**Ethan** and his friends are talking about **William** together. "He's so white he looks like a ghost," says **Duante**. "I've already been to his house because his mother is friends with my mother", says **Daniel**. "Oh yeah? What's it like at his place?", asks **Ethan**. "I bet it looks like a haunted house!", laughs **Duante**. "It's true that there are some weird things", answers **Daniel**. "For example, his older sister stays in her room all the time, and you never see her. "That's creepy, she must be a ghost too!" exclaims **Duante**. "What else?" "Well... there's some strange stuff hanging on the walls too", says **Daniel**. "Argh, it sounds like a horror movie!", exclaims **Ethan**. "Tell us more!" **Daniel** enthusiastically adds some disturbing details to satisfy his friends. At a certain point, the stories **Daniel** tells become so unbelievable that it's obvious he's making it up, but the three boys pretend to believe it to keep the fun going. That night, as he goes to sleep, **Ethan** thinks to himself that he never had so much fun before meeting his new friends.

The days pass and the day of the museum visit arrives. The students are delighted: Mr. **Brown** has explained to them that they are going to do a treasure hunt in the museum in small groups of three or four. From their different seats in the bus, **Ethan**, **Duante** and **Daniel** smile and wink at each other: the three of them will definitely team up for the game. The bus arrives at the museum and all the students get off. Mr. **Brown** asks them to form their groups. The students organize themselves and, not surprisingly, the only one who ends up on his own is **William**. "Who would like to take **William** in their group?", asks Mr. **Brown**. No one raises a hand and some students snicker. "If that's how it's going to be, I'll choose. **William**, you will go with **Ethan**, **Duante** and **Daniel**", announces Mr. **Brown**. The three boys are startled, then look at each other grimacing. "Oh no...", whispers **Daniel**.

The game starts and the groups go in different directions. The group of four boys starts walking. **Duante** is holding the map. The museum is huge and full of corridors in all directions: a real labyrinth! None of them speak and the atmosphere is rather tense. "Well", says **Duante**, reading the instruction sheet given by Mr. Brown, "first we have to go to the herbivore room and there we have to find out what the biggest dinosaur in the room is called. We'll follow the arrows to the right room." Without enthusiasm, the four boys start walking in silence. **Ethan** feels desperate: what was supposed to be a great day is turning into a nightmare. There's no way his new friends are going to have a bad time because of **William**. **Ethan** catches up with **Duante**, who is walking ahead, and whispers to him, "We have to do something. We can't let him ruin our day." "But what do you want us to do?", asks **Duante**. "We'll lose him in the museum!", replies **Ethan**. **Duante** opens his mouth and looks at him with round eyes: "Brilliant! Tell Daniel: when we arrive at the room, we'll let him walk ahead of us, then we'll run away!" Very excited, **Ethan** joins **Daniel** and whispers his plan to him. **William** is walking at a few meters behind them. **Daniel** seems a little hesitant. "We’ll just have to say that he got lost ", **Ethan** reassures him.

In the meantime, the little group has almost arrived in the herbivore room. **Duante** can hardly stop himself from laughing. **Ethan**, on the other hand, stays focused. He takes his plan very seriously. **William** points to a dinosaur and announces in his piercing voice, "It's that one, that's the biggest dinosaur in the room!". "Well, let's go, the first one to arrive wins!", says **Duante,** laughing. **William** rushes into the room, dodging round the numerous groups of visitors. "Now, quick, let's go!", says **Ethan**, running in the opposite direction. The two boys follow him down the corridors laughing. A few hundred meters later, short of breath, they stop behind a sign. "Is it OK, he didn't follow us, did he?", asks **Daniel**. "Not likely, he was heading straight for the dinosaur to get there first! He's probably only just realized that we aren't there anymore!", answers **Ethan**. **Duante** doubles up with laughter and has to lean against the wall to stop himself from falling over: "I can't stop imagining his face when he realizes that he’s lost, it's so funny!" **Ethan** is delighted with the success of his idea: thanks to him, his friends will have a good day.

The three friends spend the afternoon having fun together in the museum, and then it's time to meet the rest of the class to go trampolining. **Ethan**, **Duante** and **Daniel** hurry out of the museum to get back to the bus on time. **William** is sitting alone a few meters away from the rest of the class. His eyes are red and swollen.

After a short bus ride, the students arrive at the trampoline park. In the hall, large trampolines are surrounded by safety nets with an open space for getting in. Delighted, the students hurry to remove their shoes and line up. "It's a maximum of three per trampoline!", the instructor announces, letting the students in as they come. **Daniel** finds himself on a trampoline with the two students who were before him in the line. **Duante** and **Ethan** will be together with... **William**, who, by coincidence, was just behind them. "Come on, that's not possible!", grumbles **Duante**. As he starts to jump on the trampoline, **Ethan** is thinking fast: he can't let **William**'s presence spoil his friend's fun. "I'm going to push him until he leaves!", says **Ethan** to **Duante**. **Ethan** leaps across the trampoline and approaches **William**, who is standing near the space without a safety net. **Ethan** glances at **Duante**, who is watching him intently. "It's okay to do this since it's to protect my friends", **Ethan** says to himself as he reaches out to push **William** with both hands toward the opening in the net. **William** is caught off-guard. He tries to grab at the net, then loses balance and topples backwards, right through the opening. Mr. **Brown**, who is on the floor, shouts and rushes over just in time to catch **William** before he hit his head on the floor. "Are you crazy, **Ethan**?" shouts Mr. **Brown**. "You get down immediately!", he yells.

Mr. **Brown** may be a very nice teacher, but when he gets angry, he gets really angry. He yells at **Ethan** for a good ten minutes before sending him to the bus until the other students finish the activity. All alone on the bus, **Ethan** keeps repeating to himself, "I did it to help my friends, so I had the right to do it." When the students get back to the bus, **Duante** and **Daniel** hurry to sit next to **Ethan**: "What did he say?" "He's going to punish me," **Ethan** replies. "No swimming pool for me for three weeks."

The days pass and what happened is gradually forgotten. **Daniel**'s birthday is approaching and the three friends are already looking forward to a great day together. One Monday morning, **Daniel** comes to school with bad news. His mother and **William**'s mother, who are friends, have seen each other over the weekend: "My mother has had the idea of inviting **William** to my birthday party." "No!" exclaims **Duante**.

"I told her we didn't like him, but she wants me to make an effort...", explains **Daniel**. "The poor thing has no friends!", he adds, rolling his eyes and imitating his mother's voice. "Our day's been ruined," says **Duante**. "What do you think, **Ethan**?", asks **Daniel**. "This time it's really war", says **Ethan**. "What are you going to do?", asks **Duante**. "It's simple, we have to make him not want to come anymore. We'll do everything we can to put him off." "Everything?" asks **Daniel**, a little worried. "We're not going to hit him, are we?" "Yes, why not? You can do that to protect your friends, and I want to protect my friends," answers **Ethan**.

**Story 3**

**Lisa** is in Grade 4/5/6. At school she is very nice to everyone and always in a good mood. **Lisa** also has something a little unusual: she was born with a cleft palate, which is sometimes called a "harelip". This means that when she was in the womb, her upper lip didn't have time to form properly before she was born. **Lisa**'s lip looks like it's been cut in half, which makes her mouth look funny. There is an operation that can fix her lip, but the doctors want to wait until she has finished growing before they operate. This bothers **Lisa** a little, who would like to have a mouth like everyone else, but after all, she's used to it now, and so are her friends.

At the beginning of the year, a new student, **Alan**, arrives in the class. When she sees him, **Lisa** thinks he has beautiful eyes. So, at recess, she goes up to him and asks him to play with her and her friend, **Najwa**. But when she comes up to him, he looks at her and says, "Yuck. You're so ugly! With your mouth, you look like a rabbit!" **Lisa** is stunned: since she's been at this school, it's the first time anyone has made a comment about her mouth! At home in the evening, her parents can see that she is out of sorts. But **Lisa** isn't the type to complain, so she doesn't tell them what has happened.

The problem is that the next day, at lunch, **Alan** says in front of the whole class that he doesn't want to sit across from her while he eats because it will spoil his appetite, which makes some students laugh. **Lisa** is so upset by his remark that she scarcely touches her food. At recess, her friend **Najwa** can see that she is sad. "Do you want me to go talk to him?", she asks. **Lisa** hesitates for a few seconds and then nods. **Najwa** then heads to the soccer field where **Alan** is playing with other students in the class. A little nervous about interrupting their game, she beckons **Alan** over and then says, "Er... That wasn't very nice what you said to **Lisa** earlier!" **Alan** looks at her, then shrugs and replies, "If she's mad about that, then she has no sense of humor!". "That's right!" says another boy who is on the field. "It's not my fault she has a rabbit's face!", adds **Alan**, laughing. The students around laugh. "Don't you agree, **Najwa**?", he asks her. The other students have moved closer and stare at **Najwa**, waiting for her answer. **Najwa** feels uneasy and can't think of anything to say in response, so she just gives an embarrassed smile. "Hah, I knew it, she agrees!", exclaims **Alan** triumphantly, laughing again. **Najwa** goes back over to **Lisa** who asks her, "So? What did he say?" "That if you were angry about that, it shows you don't have a sense of humor", answers her friend without looking at her.

Observing **Alan** over the next few days, **Lisa** realizes that he is very popular with the other students: everybody seems to think he's great and he already has a lot of friends even though he has only just arrived! In fact, it's true that with the others, **Alan** is very nice: he helps them, he always comes up with good ideas for games and he's funny. He's also very handsome and has a knack for complimenting the girls. When he plays soccer, the girls in the class come and hang out near the field to watch him, waiting for him to call out, "Hi, beautiful!" or "Nice dress!", which makes them laugh and blush. In fact, it's only with **Lisa** that **Alan** is different. Every day, **Lisa** hopes that he will change his attitude and that he will treat her the same way as the others. So, during recess, she passes by the soccer field from time to time, hoping that he will say something nice to her too. But all she gets is a "Hey, rabbit! Go show your dirty mouth somewhere else!".

One day, **Lisa** hears another boy ask **Alan,** "But why are you being mean to her?". **Alan** replies, "But it's her fault! She knows that if she comes here, she'll be called a rabbit! All she has to do is not come here! She just has to hide her rabbit face!"

Little by little, the other students in the class also start calling **Lisa** "Rabbit". She feels like they don't even remember that she has a name! One day, **Alan** brings a carrot to school in his schoolbag. In the schoolyard, accompanied by two other boys, he puts it under **Lisa**'s nose, shouting, "It's rabbit food, you must like it! So go ahead and eat it!" **Lisa** backs away, but **Alan** insists, so she ends up running away, chased by the three boys. She only manages to escape them by hiding in the girls' bathroom.

Now, at lunch, almost everyone in the class acts disgusted when she sits across from them. **Lisa** is so uncomfortable that she no longer touches her plate. After three weeks, **Lisa**'s teacher, Mrs. **Garcia**, realizes that she is not eating lunch. Worried, she calls **Lisa**'s parents to let them know. When **Lisa** gets home in the evening, her parents ask her a lot of questions: "What's going on at school?", "Why aren't you eating anymore?", "Tell us, honey!" **Lisa** then starts to cry and explains everything to them. Very shocked, her parents decide to call the school principal to tell her what their daughter is going through.

The next day, the principal suddenly comes into the classroom and asks **Alan** to come with her to her office. The other students are intrigued and whisper among themselves, wondering what is going on. **Lisa** stares at her desk without daring to look up to watch **Alan** leave the classroom. When they arrive in the office, the principal tells **Alan** very sternly that she is aware of the comments he is making to **Lisa** about her mouth and asks him to explain. "But it's not my fault if she's weird!", protests **Alan**. "All she's got to do is be like everyone else!"

**Story 4**

**May** is in Grade 4/5/6. Since she was little, she has always been the tallest; in her class, she is even taller than the boys. **May** has also always been very beautiful, and has become more and more so as she has grown older. Her height and beauty often impress the other students and many of them look up to her. Even her two best friends, **Jennifer** and **Ashley**, are sometimes afraid of her and would rather go along with what she says than risk an argument. **May** is well aware of the power she has over others and sometimes she takes advantage of it. This year especially, since her parents told her they were getting divorced, she has become very spoiled and bossy with the other students in her class.

**Joshua** has been in the same class as **May** for three years. He is a rather quiet student who doesn't attract attention. He doesn't like arguments and prefers to find solutions so that everyone can agree when there is a problem. **Joshua** doesn't usually have much to do with **May** because she plays with her friends at recess, while he is a big fan of basketball and plays every day with other students. But this year, since the beginning of the school year, they have been sitting next to each other in class, and **Joshua** is irritated by **May**'s behavior. In class, she talks all the time to **Ashley** whose desk is right behind her, which prevents **Joshua** from concentrating. And above all, it annoys him to see **May** acts as if she is the queen of the world.

One Friday afternoon, to congratulate them for having worked hard all week, their teacher, Mr. **Davis**, announces to the class that they can have some quiet fun for the last half hour before school ends. **Joshua** thinks he will be able to finish the comic book he has started to read. As he gets it out of his desk, **May** announces to everyone that she wants to hold a class beauty contest! She orders the students to each write down the name of the girl they think is the most beautiful in the class on a piece of paper. Some of the students are thrilled with the idea and start to think about who to pick, but **Joshua** can see that others aren't too keen on it, even though they don't dare to say anything. Everyone knows that **May** wants to hold this contest because she thinks she will win. Deciding that there is no way he will play her game, **Joshua** opens his comic book and starts to read without taking any further notice of **May**.

After a few minutes, she orders everyone to give her their piece of paper. When she gets to **Joshua**, she asks him, "Where is your paper?". **Joshua** pretends he didn't hear her and continues reading. "Hey, are you deaf?", exclaims **May**, and when she doesn't get an answer, she takes the comic book out of his hand to stop him from reading. "Give it back!", snaps **Joshua**, "You have to say which girl in the class you think is prettiest first", replies **May**. "I don't have to play your game", he exclaims. "Yes, you do!", she replies. "We all know that you want to do this to win, but actually you're ugly!", shouts **Joshua**. As soon as he says these words, **Joshua** regrets them. He's not the type to get mad at people, let alone say mean things to them. But **May** has really pushed him to the limit. A short silence falls over the class: no one says a word and everyone stares at **May**, waiting to see her reaction. For a second, **May** is stunned - no one has ever dared to stand up to her before - then she stands up straight, looks **Joshua** over from head to toe, and says, "Who cares what you think, you little loser! Do you think we care what you think, you dwarf?" At that moment, the bell rings and all the students hurriedly grab their things to leave the classroom. **Joshua** leaves the classroom last, a little shaken by what just happened. On his way out of the school, he sees **May**, **Jennifer** and **Ashley** looking furiously at him.

Over the weekend, **Joshua** doesn't have much time to think about what happened on Friday. On Saturday, his parents take him and his sister kayaking down the river and on Sunday, he spends the whole afternoon with his grandparents', whom he adores. That night, in bed, he decides to apologize to **May** the next morning. After all, no matter how annoying she is, he didn't mean to hurt her.

On Monday morning, **Joshua** arrives at school full of good intentions. But when he gets to his desk, he discovers that someone has written "LOSER" on it in big letters with a permanent marker. Stunned, **Joshua** stands still for a moment and stares at his table. Then he looks around and sees **May**, **Ashley** and **Jennifer** in a corner of the classroom, staring at him with and smiling slightly. **Joshua**, not knowing how to react, sits down without saying anything and doesn't look once in the direction of **May** during the class.

At recess, he sees **May** in the distance talking and smiling with two boys from his class with whom he usually plays basketball. When he gets to the court, one of the boys reaches out to block his path and says, "You're not playing with us. We don't take dwarves on our team." "Too bad for you", **Joshua** replies, offended, before walking away. As he walks around the schoolyard, he thinks about his height. It's true that he's not very tall, but he's never really thought about it until now. In the afternoon, **May** isn't in class because she has a doctor's appointment. "At least I'll be safe", **Joshua** thinks. But during class, in the middle of a dictation, a ball of paper suddenly hits his head and rolls onto his desk. Surprised, **Joshua** picks it up and sees that there is something written on the paper. He unfolds it and reads, "You suck, dwarf". **Joshua** freezes. **May** isn't there, so it can't be her. **Ashley** and **Jennifer** are too far away in the class to have been able to throw it at him and anyway, it's not their style to do that. But that means it must be someone else. **Joshua** feels his throat tighten. He can understand that **May** is angry with him. And **Ashley** and **Jennifer**, since they are her best friends. But it seems that other students in the class have decided to take it out on him for what happened last Friday. This was something he wasn't expecting at all, as he has never had problems with the others at school.

The next day, **May** is back. When she arrives at class in the morning, **Joshua** goes straight to her and says, "I'm sorry I said you were ugly, I didn't mean it. I apologize.". **May** gives him a scornful look and says, "It's too late. You should have thought about that before, you dwarf!" "Why do you keep calling me that?" "It's not just me who calls you that, the others do too. Everyone calls you 'dwarf' in case you haven't noticed!" **Joshua** feels disheartened and goes and sits down in his place. **Ashley**, who heard the exchange, remarks to **May**, "He looks quite sad... ". "So what?", retorts **May**. "Perhaps you could be nicer to him?", suggests **Ashley**. "My parents are getting divorced, remember? So it's not my fault if I'm not nice!", answers **May**.

From now on, since the other boys don't want him to play basketball with them, **Joshua** spends his recesses alone. He hangs out in the corridors or the bathroom to pass the time. Now, when he looks in the mirror, he thinks he's small, whereas he has never worried about his height before. Sometimes he even feels like he's shrunk since people have started calling him a dwarf. As the days pass, **Joshua** thinks that maybe **May** has calmed down and will stop bothering him. Then, one day, when he returns to class after recess, **Joshua** finds an envelope in his schoolbag. Very surprised, he opens it and discovers inside a sheet of paper on which several insults are written: "Loser", "Dirty dwarf", "You're too small", and so on, with, underneath, the signatures of all the children in the class. Shocked, **Joshua** feels so hurt that he wants to cry. When **May** sits down next to him, with a big smile on her face, he asks, "Why are you doing this?" "Stop saying it's me!", exclaims **May**, annoyed. "You can see that it's everybody: everybody has signed!"

**Story 5**

**Rachel** is in Grade 4/5/6. She is a good student, but she is also very quiet and shy. In class, nobody really notices her. This doesn't bother her much because she is used to being alone and, to occupy herself, she often imagines incredible stories in which she has fabulous adventures. Her teacher, Mrs. **Thomas**, kindly says that she is a great dreamer who always has her head in the clouds.

This year, **Rachel**'s class is going on a school trip to the seaside for a week. **Rachel**, who loves the sea, is delighted. She is already imagining all the stories of hidden treasure, pirates and magical mermaids that she will dream about. In the bus on the big day, all the students are very impatient to arrive and Mrs. **Thomas** has a hard time keeping them calm. Moreover, it seems that a class of students of the same age from another school will be staying in the center at the same time as them. Everyone wonders what the other class will be like and if they will be nice. Some of the girls in the class are already imagining meeting their Prince Charming and are laughing with each other during the whole trip imagining the encounter. **Rachel**, on the other hand, doesn't care at all and is already off in her daydreams of adventure.

When they arrive, the students settle into their different rooms and go to the dining hall to meet the other class. The two teachers have known each other for a while and are very happy to see each other. In the other class, one boy in particular attracts the attention of the girls in **Rachel'**s class. His name is **Mehdi** and he is very handsome. Even **Rachel**, who usually doesn't pay attention to such things, notices how handsome **Mehdi** is.

The first day passes quickly. The students visit the center and take a walk to the beach, then go home for dinner and go to bed. The next day, the two teachers suggest that the students of both classes go on an activity together. The students are very happy about this, and quickly get to know each other. Two girls from **Rachel**'s class, **Maria** and her best friend, **Janet**, stick close to **Mehdi**. This upsets **Tyrell**, a boy in their class, who would like **Maria** to be interested in him instead. **Rachel**, as usual, remains isolated and lost in her thoughts. The rest of the week goes smoothly and the two classes get along very well. However, **Mehdi** seems to be a little tired of being followed everywhere by **Maria** and **Janet**, who don't let him out of their sight.

On the last evening, a party is planned to celebrate the end of the stay. There will be chips, music and lights: everything is planned and the atmosphere is really festive. Everyone puts on their best clothes for the occasion. The girls in **Rachel**'s class are extremely excited. All of them would like to invite **Mehdi** to do a slow dance with them, but none of them dare to, because **Maria** has made it clear to everyone that **Mehdi** is hers and that anyone who tries to touch him will regret it. In the class, **Maria** is someone no one really dares to stand up to. The evening begins and everyone is having fun. **Rachel**, in a corner, absent-mindedly watches the others dance while munching on peanuts. Suddenly, she feels someone tapping her on the arm. Turning around, she finds herself facing **Mehdi**, who smiles at her and asks her to dance with him. Surprised, **Rachel** stares at him at first without answering. **Mehdi** takes her hand and leads her with him to the middle of the room, then starts to dance. Out of the corner of her eye, she sees **Maria**, **Janet** and some of the other girls in the class staring angrily at her. Without paying much attention, **Rachel** lets **Mehdi** guide her and enjoys the dance.

It is getting late and the teachers turn off the music and send everyone to bed, despite the students' complaints. That night, in **Rachel'**s dreams, the pirate looking for treasure looks a bit like **Mehdi**. On Saturday morning, her parents pick her up from the bus and **Rachel** goes home.

On Sunday, she and her parents go to dinner at her grandmother's house in the country. **Rachel** meets her cousins and plays with them in the garden all afternoon. In the evening, in the car, she dozes during the return trip. Suddenly, she feels her phone vibrate in her pocket, once, then a second time. She is surprised because she never usually receives messages at the weekend. She takes the phone out of her pocket and opens the messages. They are from an unknown number and say, "Die" and then "Idiot". **Rachel** stares at her phone and reads the messages three times to make sure she is not dreaming. She really doesn't know what to think. She looks up at her parents, but they are in the middle of a discussion, and she doesn't want to interrupt them. Besides, her parents didn't like the idea of her having a phone, and **Rachel** had to beg them for months to give her one for her birthday. "If I show them the messages, maybe they won't want me to have a phone anymore," she thinks. She puts the phone back in her pocket. "Maybe it's a mistake", she thinks, not quite convinced.

On Monday, when she arrives at school, **Rachel** has forgotten about the messages. During the day, her phone stays switched off in her bag. At noon during recess, **Rachel** switches it back on to see if her mother has sent her anything. Three new messages appear on the screen, but **Rachel** suddenly feels her breath catch when she sees that they are from the same unknown number as the day before. After a few seconds, she decides to open them. "I'll poke your eyes out", says the first one. The second one just contains insults and the third one, a small skull and crossbones. Slowly, **Rachel** writes, "Who are you?", then, after a brief hesitation, pushes the "send" button. She waits for a few minutes, then, just as she is about to put her phone back in her bag, a new answer from the unknown number appears on the screen: "Your worst nightmare". In the afternoon, **Rachel** cannot concentrate in class. She is preoccupied by the unknown number and threatening messages. She is waiting for the day to end so she can turn on her phone and check for more messages. That evening, when she gets home, **Rachel** finds more messages, but this time from a different number. Again, they are insults and threats.

In her bed, **Rachel** can't sleep. She has left her phone on the bedside table and stares at it without closing her eyes. After a while, she decides to send an answer to the two numbers: "Leave me alone", she writes. As if they were waiting for this, the two strangers immediately reply, laughing and making fun of her. One of the messages says, "We will never leave you alone." After several hours, **Rachel** finally falls asleep. During the night, she has a horrible nightmare. At one point, she wakes up with a start. She looks at her alarm clock: it is 5 o'clock in the morning. Afraid of having another nightmare, she decides to wait in the living room for her parents to get up rather than go back to sleep.

When she arrives at school, **Rachel** looks really bad because of her short night. "Have you seen her face?", whispers **Tyrell** to **Maria** and **Janet** as **Rachel** walks past them to the classroom. "It's perfect, she won't be dancing with boys anymore with a face like that", **Maria** replies with a laugh. "My cousin showed me an app that lets you schedule messages to someone hours in advance", says **Tyrell**. "We could use it to send messages to her all night long." "Great!", replies **Maria**. **Tyrell**, delighted that **Maria** likes his idea, promises himself to try and think of other ideas so that **Maria** will finally take an interest in him. **Janet**, in a timid voice, remarks: "But isn't sending messages at night going a bit far?" "Of course not, why do you say that?", answers **Maria**. "It's only messages, it’s not going to kill her!", adds **Tyrell**, who hopes to impress **Maria**.

The days go by and things are getting worse and worse for **Rachel**. A third unknown number has been added to the two others and she receives dozens of messages a day. She looks as pale as a ghost and has huge dark circles under her eyes from staying awake at night in fear of receiving new messages. Now she receives insulting messages almost all the time, even during the day. In class, she keeps her phone on silent mode on her lap hidden under her desk to check the messages she receives throughout the day. Her teacher, Mrs. **Thomas**, has noticed that she is not listening to anything in class, but since **Rachel** has always been a very good student, it doesn't really affect her grades, so Mrs. **Thomas** leaves her alone.

On one occasion, **Rachel** tries switching off her phone all day without looking at the messages she has received. The problem is that when she switches it back on in the evening, she receives more than sixty messages in one go. This cascade of insults and threats seems even worse than receiving them one by one during the day. One morning, when she reads the latest message she has received, **Rachel** can't take it anymore and burst into tears in the middle of the classroom. The other students are stunned. Mrs. **Thomas** tries to get her to tell them what is wrong and to comfort her, but **Rachel** can't get a word out through her tears. Not knowing what to do, Mrs. **Thomas** suggests that she goes to the infirmary to rest until recess.

When they meet in the schoolyard, **Janet** says to **Tyrell** and **Maria,** "Have you seen how she was crying? We have to stop, we've done enough now!" "Oh come on, it's okay, just because she's crying a little doesn't mean it's super serious", **Tyrell** replies. "It's just messages, it doesn't really hurt!", he adds, turning to **Maria** to see her reaction. After a second of silence, **Maria** exclaims, "Well, I'm sure it doesn't upset her. In fact, I'm sure she's just happy to be getting attention and interest." "Yeah, that's right, she has almost no friends at school, at least we talk to her!", adds **Tyrell**.
